# Involvement of Gut Microbiota in the Development of Psoriasis Vulgaris

**DOI:** 10.3389/fnut.2021.761978

**Published:** 2021-11-22

**Authors:** Chaonan Sun, Ling Chen, Huan Yang, Hongjiang Sun, Zhen Xie, Bei Zhao, Xuemei Jiang, Bi Qin, Zhu Shen

**Affiliations:** ^1^School of Medicine, University of Electronic Science and Technology of China, Chengdu, China; ^2^Department of Dermatology, Daping Hospital, Army Medical University, Chongqing, China; ^3^Institute of Toxicology, School of Military Preventive Medicine, Army Medical University, Chongqing, China; ^4^Department of Ophthalmology, Sichuan Academy of Medical Sciences and Sichuan Provincial People's Hospital, Chengdu, China; ^5^Department of Dermatology, Institute of Dermatology and Venereology, Sichuan Academy of Medical Sciences and Sichuan Provincial People's Hospital, Chengdu, China; ^6^Acupuncture & Moxibustion Research Institute, Sichuan Academy of Traditional Chinese Medicine, Sichuan Second Hospital of Traditional Chinese Medicine, Chengdu, China

**Keywords:** psoriasis, gut microbiota, 16S rRNA sequencing, gastrointestinal symptom, fecal microbiota transfer

## Abstract

**Objectives:** Psoriasis is a common chronic recurrent dermatitis. Accumulating observations show gut microbiota dysbiosis in psoriasis. We intend to further investigate the relationship between intestinal microbiota and psoriasis development.

**Design:** We first performed an epidemiological investigation on differences of gastrointestinal discomfort symptoms between patients with psoriasis and general population. Then variation of gut microbiota in patients with psoriasis (un)treated with acitretin plus narrow-band ultraviolet B (NB-UVB) was analyzed by 16S rRNA sequencing. We last compared recovery status and vital cytokines (lesion and intestine) of mouse psoriasiform models, which were transplanted with fecal microbiota from patients with psoriasis or healthy controls.

**Results:** (1) About 85.5% of patients with psoriasis vs. 58.1% of healthy controls presented with at least one gastrointestinal symptom. The prevalence of investigated symptoms (e.g., abdominal distension and constipation) were significantly higher in patients, compared with controls (*p* < 0.05). Passing flatus and constipation were significantly correlated with psoriasis (*p* < 0.05 in both cases). (2) The abundance of Ruminococcaceae family, *Coprococcus_1* genus, and *Blautia* genus were decreased with psoriasis improvement (*p* < 0.05, respectively), which had been demonstrated significantly increased in psoriasis. (3) Mice receiving psoriatic microbes transplantation showed delayed recovery of psoriasiform dermatitis and less reduction of interleukin (IL)-17A than those receiving healthy microbiota or blank control (*p* < 0.05 and *p* < 0.01, respectively).

**Conclusion:** Multiple evidence we provided here preliminarily demonstrates the involvement of gut microbiota in the different degree of psoriasis activity. The strategy based on overall microbial communities is expected to be a promising supplementary for long-term management of psoriasis.

## Introduction

Psoriasis is a common chronic skin inflammation, and it can even cause systemic involvement for those with early-onset and severe conditions (1). Although the exact pathogenesis is not completely known, psoriasis has been considered a relapsing-remitting disease triggered by environment–immunity interaction in genetically susceptible individuals.

Treatment options have advanced following deeper understanding of the pathophysiology of psoriasis, e.g., interleukin (IL)-23/IL-17-targeted agents. However, a survey from the National Psoriasis Foundation reveals widespread treatment dissatisfaction in patients with psoriasis (52.3%) (2), especially in reducing the recurrence and managing its long-term chronic course.

The gut microbiota, 100 trillion microorganisms residing in the human gastrointestinal tract, has been documented to provide essential benefits to host health, particularly by orchestrating immune/inflammation homeostasis (3). Recently, the concept of gut–skin axis has linked the disordered gut microbiome and skin diseases through a network of inflammatory mediators (4, 5). Evidence suggests that lower gut microbiome diversity is associated with higher levels of fat and low-grade chronic inflammatory process (6). Dysbiosis in microbial communities has been implicated in continuous immunological stimulation, as a trigger for local and (or) systemic immune responses, including inflammatory bowel disease (IBD) and allergy (7, 8).

Accumulating evidence has suggested the association between dysbiosis of gut microbiota and psoriasis (5). (1) The epidemiological association between psoriasis and IBD showed increased prevalence of IBD in patients with psoriasis, and *vice versa* (9, 10). (2) The partial shared susceptibility loci and DNA polymorphisms between psoriasis and IBD (e.g., 6p21.3) further supports their association at genetic level (11, 12). (3) Notably, patients with psoriasis have been shown decreased bacterial diversity and changed relative abundance of certain bacterial taxa, resembling dysbiosis in IBD (13–17). (4) The microbiota profile in severe psoriasis has been demonstrated different from those with mild one (18). By now, elucidating gut microbiota status and the cross-talk of microbiota and immune system in patients with psoriasis are at their initial stages. It will provide theoretical basis to develop promising microbiome-based therapeutic options.

**Figure 1:**
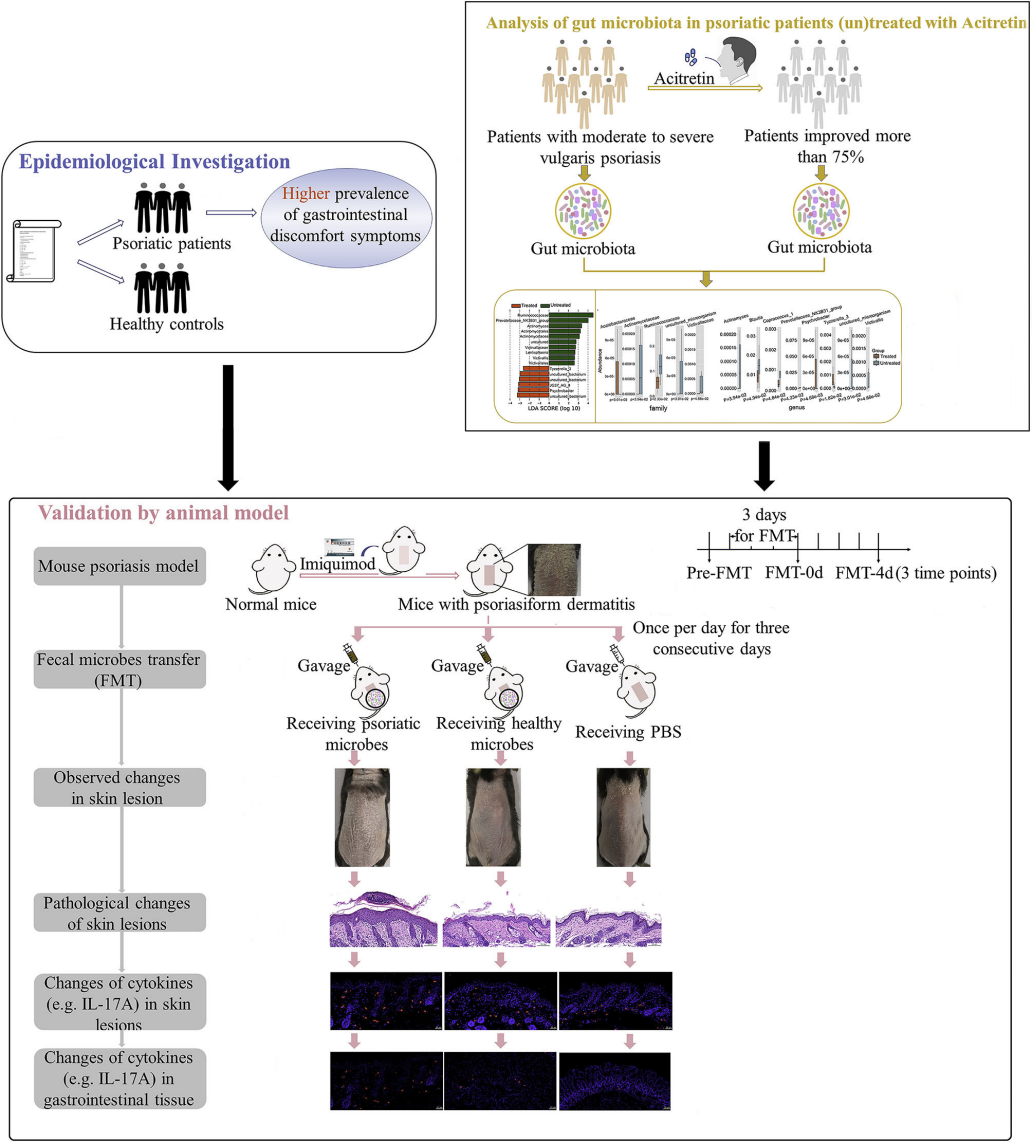
Study workflow. Epidemiological investigation on differences of gastrointestinal discomfort symptoms between patients with psoriasis and general population were first performed. Then variation of gut microbiota in patients with psoriasis (un)treated with acitretin plus NB-UVB was analyzed by 16S rRNA sequencing. Last, experiment with mouse psoriasiform models receiving fecal microbiota transplantation (FMT) was performed for crucial results validation. Visual observation and samples collection were performed at pre-FMT, day 0 after FMT (FMT-0d) and day 4 after FMT (FMT-4d). NB-UVB, narrow-band ultraviolet B.

The purpose of the current study is to further explore the correlation between gut microbiota of patients with psoriasis and the degree of disease activity by epidemiological investigation of gastrointestinal discomfort symptoms in patients with psoriasis, and by the analysis of gut microbiota variation with psoriasis improvement. And we also analyzed the recovery status and pathogenic cytokines (e.g., IL-17A) in mouse psoriasiform models that were transplanted with fecal microbiota from patients with psoriasis or healthy controls (Figure 1). Multiple evidence we provided here preliminarily demonstrated the involvement of gut microbiota in psoriasis development. The strategy by manipulating overall gut microbes is expected to be a promising complementary therapeutic method for the long-term management of psoriasis.

## Materials and Methods

### Ethical Statement

All human experiments and animal experiments were approved by the Ethics Committee of Sichuan Provincial People's Hospital. Written informed consent of all patients with psoriasis and healthy controls was obtained.

### Epidemiological Investigation

This epidemiological survey was performed from January 2018 to June 2020 to investigate the prevalence and severity of gastrointestinal discomfort symptoms in patients with psoriasis and general population by the questionnaire (Supplementary Appendix 1). All participants were all inhabitants of the Sichuan Province, China (western China). The inclusion criteria for psoriatic patents and healthy controls are: 18–60 years old; traditional Chinese diet; psoriasis diagnosed by at least one dermatologist (for patients). The exclusion criteria are: using antibiotics or immunosuppressive agent within 1 month; long-term use of probiotics or prebiotics; long-term consuming yogurt, pickles, or cheese; pregnancy; a history of acute/chronic gastrointestinal infection, gastrointestinal pathology, or gastrointestinal surgery; a history of arthritis, enthesitis, or dactylitis; and current extreme diet (e.g., vegetarian, parenteral nutrition, or macrobiotic diet).

### Patients and Fecal Samples

#### Patients With Psoriasis (Un)treated With Acitretin Plus NB-UVB and Their Fecal Sample Processing

Patients with moderate-to-severe vulgaris psoriasis from outpatient clinic of the Department of Dermatology were included. The general inclusion and exclusion criteria were the same as the patient part of the epidemiological investigation. Patients in group with acitretin treatment had been orally administered with acitretin capsules for 1 month at a dose of 0.5 mg/kg/day (Huapont Pharmaceutical Co., Ltd, Chongqing, China) plus narrow-band ultraviolet B (NB-UVB) once every 2 days for 1 month. They were all improved more than 75% based on their psoriasis area and severity index (PASI) score. Untreated patients with psoriasis with matched age and gender served as controls. Fecal samples of all patients were collected in the morning, and immediately stored at −80°C for 16S rRNA analysis. The whole collection procedure was completed within 30 min.

#### Participants and Their Fecal Sample Processing for Fecal Microbial Transplantation

Fecal samples were collected from four patients with moderate-to-severe psoriasis (two male participants and two female participants) and four age- and gender-matched non-psoriatic controls. All these voluntary participants were 18–45 years old, and other requirements are in accordance with the inclusion and exclusion criteria of epidemiological investigation. Their stool samples in the morning were freshly collected three times within 1 month, for obtaining a representative inoculum. These samples were free of enteropathogenic bacteria, viruses, and parasites. About 20 g of mixture of the three repetitions from each participant was divided into five aliquots after removal of undigested solids within 30 min of collection. Each 0.5 g was stored in a sterile storage tube at −80°C.

#### Processing Mouse Fecal Sample for 16S rDNA Sequencing Analysis

All animal experiments were conducted in accordance with National Institutes of Guidelines for Animal Care and Use. In order to confirm the successful fecal microbial transplantation (FMT), characteristics of mouse gut microbes before and after FMT were analyzed by 16S rDNA sequencing technology. Following slightly pushing mouse lower abdomen using a moist cotton swab to provoke defecation in the morning, a minimum of five fresh fecal pellets were collected in sterile storage tubes and immediately kept at −80°C. The feces were collected before FMT (pre-FMT), at 0 day and 4th day after complete FMT procedure, respectively.

### DNA Extraction and 16S rRNA Amplification Sequencing Analysis

DNA was isolated from human and mouse samples using standardization cetyl-trimethyl ammonium bromide (CTAB) methods (19) and QIAamp 96 PowerFecal QIAcube HT kit (QIAGEN, Hilden, Germany) following the instructions of the manufacturer, respectively. The amplifications of V4 (human samples) and V3 and V4 (mouse samples) regions of bacterial 16S rRNA gene were performed by PCR using the barcoded primers of 515F and 806R (human samples) and 343F and 798R (mouse samples), respectively. Amplicons were further purified with GeneJET Gel Extraction Kit (Thermo Scientific, Waltham, MA, USA) and pooled together. All purified samples were sequenced on the Illumina Miseq platform (Illumina Inc., San Diego, CA, USA) with generating 300-bp paired-end reads.

### Microbial Profiling Analysis

All raw sequencing data were in FASTQ format. Trimmomatic software (Jülich, Germany) was used to trim raw sequence that cutting off ambiguous bases and base quality below 20 found after sliding window trimming approach (20). Contiguous sequences were then assembled by FLASH software (Charlestown, MA, USA) (21). Operational taxonomic unit (OTU) tables with 97% nucleotide identity were constructed under the condition that sequences were performed further denoising using QIIME software (version 1.8.0, Boulder, CO, USA) (22). The representative read of each OTU was annotated and blasted against Silva database (Version 123, East Lansing, MI, USA) (23).

The Shannon's diversity, Simpson diversity index, Chao1 index, and abundance-based coverage estimator (ACE) were calculated to estimate the within-community diversity and richness of the gut microbiota. Based on alpha diversity metrics, rarefaction curves were generated and drawn by “vegan” package in R (Version 2.15.3) to assess depth of coverage. UniFrac distances between bacterial communities were calculated on a phylogenetic tree, and unweighted results were represented in principal component analyses (PCoA) or non-metric multidimensional scaling (NMDS) using R software (Version 2.15.3) (24). The results of Euclidean distance were depicted in NMDS. Differentially abundant taxa between groups were identified by MetaStat analysis and linear discriminant analysis (LDA) coupled with logarithmic LDA score cutoff of 4.0 (25). MetaStat analysis was performed by using the R software (Version 2.15.3), *p* < 0.05 was set as significant threshold.

### Imiquimod-Induced Psoriasiform Dermatitis Model in Mice

The 8-week-old female C57BL/6 mice (18–20 g, Chengdu Dossy Experimental Animals, Sichuan, China, certification No. SCXK chuan 2015-030) were fed with free access to feed and water under specific pathogen-free conditions. A 2 × 4 cm area of dorsal skin of 60 mice was shaved and depilated. A daily topical dose of 62.5 mg of listed imiquimod (IMQ) cream (5%, Mingxinlidi Laboratory, Sichuan, China) was then applied on the hair-free back for 5 consecutive days to induce psoriasiform dermatitis. The severity of skin inflammation was evaluated by scores of the skin scaling and erythema (0–4, respectively) (26). About 45 mice with similar scores were selected and randomly divided into three groups.

### Transplanting Human Fecal Microbiota Into Psoriasiform Dermatitis Model

After thawed for about 15 min on ice, 2-g frozen fecal samples from patient group or healthy group (0.5 g from each donor) were pooled and diluted in sterile reduced phosphate-buffered saline (PBS, 0.1 M, pH 7.2) at 200 mg/ml. After vortex, the suspension was passed through 0.5-mm stainless steel laboratory sieves to remove large particulates and fibrous matters. Fecal suspension (200 μl) from patients with psoriasis (P group) or healthy controls (N group), or blank control (PBS, C group) were given by oral gavage into psoriasiform dermatitis mouse model once per day for 3 consecutive days.

### Tissue Collection and Hematoxylin and Eosin Staining

Mice were sacrificed by cervical dislocation under anesthesia with 1% sodium pentobarbital solution on pre-FMT, day 0 after FMT, and day 4 after FMT. The 0.5 × 0.5 cm skin lesions were gently removed and rinsed with physiological saline. They were immediately formalin-fixed (4%) and embedded in paraffin. Hematoxylin and eosin (H&E) staining was performed routinely. Epidermal thickness was evaluated under two high-power fields of light microscope (NIKON ECLIPSE CI, Tokyo, Japan) by three independent researchers.

### Immunofluorescence Studies

After routine processing and blocking, sections were incubated at 4°C overnight with anti-mouse primary antibodies against tumor necrosis factor-alpha, TNF-α (RRID: AB_2835319), interferon-gamma, IFN-γ (RRID: AB_10857066), IL-17A (RRID: AB_2838094), IL-17F (RRID: AB_2842177), and IL-23(RRID: AB_10852886), or isotype control, respectively. After rinsing, sections were treated with Cy3-conjugated goat anti-rabbit IgG secondary antibody (RRID: AB_2861435) for 2 h, and then counterstained with 4′,6-diamidino-2-phenylindole (DAPI). Image acquisition was performed with a digital slide scanner (3DHISTECH, Budapest, Hungary) under ECLIPSE TI-SR fluorescent microscope (NIKON, Tokyo, Japan). Positive immune cells and their values were determined to assess inflammatory changes.

### Statistical Analysis

Numerical results are expressed as median with a 95% CI. Categorical variables were described with numbers and percentages. Differences of body mass index (BMI) and age between two groups were compared with Mann–Whitney *U*-test. The proportions among patients and controls were compared by chi-squared test. The relationship between gastrointestinal symptoms and psoriasis was evaluated by a logistic regression test. *P* < 0.05 was considered as a statistically significant difference.

### Data Accession

Raw sequence data of gut bacterial 16S rRNA of patients with psoriasis (un)treated with acitretin and mice with psoriasis-like dermatitis are available in the Sequence Read Archive (SRA), respectively, under the accession number PRJNA743747 and PRJNA741854.

## Results

### Higher Incidence of Gastrointestinal Discomfort Symptoms in Patients With Psoriasis Than Common Population

Totally 459 participants returned their questionnaires, and 133 were excluded because their questionnaires were incomplete. After exclusion of incomplete questionnaires, 326 (response rate 71%) were qualified for further analysis, including 159 patients with psoriasis (108 males and 51 females) and 167 non-psoriatic controls (84 males and 83 females). The two groups were age-matched, and the differences in sex ratio (27) and BMI (28) were consistent with previous epidemiological findings. The summary of demographic and clinical details was described in Table 1.

**Table 1:** Demographic information and gastrointestinal discomfort symptoms in patients with psoriasis and common population.

**Characteristic**	**Total people**	**Psoriasis patients**	**Healthy controls**	***P***
***N***	326	159	167	
**Age, median (75% CI) years**	37 (30–47)	36 (30–45)	37 (28–49)	0.301
**Sex**, ***n*** **(%)**				0.001
Male	192 (58.9)	108 (67.9)	84 (50.3)	
Female	134 (41.1)	51 (32.1)	83 (49.7)	
**BMI, median (75% CI)**	22.60 (20.68–25.36)	23.71 (21.22–26.45)	22.04 (20.31–24.09)	<0.001
**Disease duration**, ***n*** **(%)**				
<3 months		9 (5.7)	NA	
≥3, and <6 months		9 (5.7)	NA	
≥6, and <12 months		4 (2.5)	NA	
≥1, and <3 years		13 (8.2)	NA	
≥3, and <5 years		18 (11.3)	NA	
≥5 years		106 (66.6)	NA	
**Severity of disease in the last 5 years**, ***n*** **(%)**				
Not affecting daily life at all		22 (13.8)	NA	
Slightly affecting daily life		95 (59.8)	NA	
Seriously affecting daily life		42 (26.4)	NA	
**Disease involvement area in the last 5 years**, ***n*** **(%)**				
<1 palm		24 (15.1)	NA	
≥1 and <5 palms		32 (20.1)	NA	
≥5 and <10 palms		37 (23.3)	NA	
≥10 palms		66 (41.5)	NA	
**Abdominal pain**, ***n*** **(%)**				<0.001
No	260 (79.8)	114 (71.7)	146 (87.4)	
Slightly	60 (18.4)	39 (24.5)	21 (12.6)	
Moderately	5 (1.5)	5 (3.2)	0 (0)	
Seriously	1 (0.3)	1 (0.6)	0 (0)	
**Type of abdominal pain**, ***n*** **(%)**				0.661
No	171 (52.5)	80 (50.3)	91 (54.5)	
Colic pain	28 (8.6)	16 (10.1)	12 (7.2)	
Dull pain	64 (19.6)	34 (21.4)	30 (17.9)	
Stabbing pain	5 (1.5)	4 (2.5)	1 (0.6)	
Cold pain	5 (1.5)	2 (1.3)	3 (1.8)	
Gas pain	27 (8.3)	12 (7.5)	15 (9.0)	
Others	26 (8.0)	11 (6.9)	15 (9.0)	
**Abdominal flatulence**, ***n*** **(%)**				<0.001
No	220 (67.5)	89 (56.0)	131 (78.4)	
Slightly	96 (29.4)	61 (38.4)	35 (21.0)	
Moderately	9 (2.8)	8 (5.0)	1 (0.6)	
Seriously	1 (0.3)	1 (0.6)	0 (0)	
**Borborygmus**, ***n*** **(%)**				<0.001
No	265 (81.3)	115 (72.3)	150 (89.8)	
Slightly	55 (16.9)	38 (23.9)	17 (10.2)	
Moderately	4 (1.2)	4 (2.5)	0 (0)	
Seriously	2 (0.6)	2 (1.3)	0 (0)	
**Gastric acid reflux**, ***n*** **(%)**				<0.001
No	238 (73.0)	92 (57.9)	146 (87.4)	
Slightly	84 (25.8)	63 (39.6)	21 (12.6)	
Moderately	3 (0.9)	3 (1.9)	0 (0)	
Seriously	1 (0.3)	1 (0.6)	0 (0)	
**Back pain**, ***n*** **(%)**				<0.001
No	236 (72.4)	94 (59.1)	142 (85.0)	
Slightly	80 (24.5)	59 (37.1)	21 (12.6)	
Moderately	10 (3.1)	6 (3.8)	4 (2.4)	
Seriously	0 (0)	0 (0)	0 (0)	
**Belching**, ***n*** **(%)**				<0.001
No	233 (71.5)	98 (61.7)	135 (80.8)	
Slightly	91 (27.9)	59 (37.1)	32 (19.2)	
Moderately	1 (0.3)	1 (0.6)	0 (0)	
Seriously	1 (0.3)	1 (0.6)	0 (0)	
**Nausea or vomiting**, ***n*** **(%)**				<0.001
No	240 (73.6)	92 (57.8)	148 (88.6)	
Slightly	77 (23.6)	58 (36.5)	19 (11.4)	
Moderately	6 (1.9)	6 (3.8)	0 (0)	
Seriously	3 (0.9)	3 (1.9)	0 (0)	
**Passing flatus**, ***n*** **(%)**				<0.001
No	227 (69.6)	87 (54.7)	140 (83.8)	
Slightly	87 (26.7)	60 (37.7)	27 (16.2)	
Moderately	10 (3.1)	10 (6.3)	0 (0)	
Seriously	2 (0.6)	2 (1.3)	0 (0)	
**Urgency of defecation**, ***n*** **(%)**				<0.001
No	246 (75.5)	102 (64.1)	144 (86.2)	
Slightly	63 (19.3)	41 (25.8)	22 (13.2)	
Moderately	15 (4.6)	14 (8.8)	1 (0.6)	
Seriously	2 (0.6)	2 (1.3)	0 (0)	
**Constipation**, ***n*** **(%)**				<0.001
No	216 (66.3)	78 (49.1)	138 (82.6)	
Slightly	94 (28.8)	66 (41.5)	28 (16.8)	
Moderately	15 (4.6)	14 (8.8)	1 (0.6)	
Seriously	1 (0.3)	1 (0.3)	0 (0)	
**Stool frequency**, ***n*** **(%)**				0.005
Once a day	205 (62.9)	90 (56.6)	115 (68.9)	
2–3 times a day	65 (20.0)	36 (22.6)	29 (17.3)	
More than 3 times a day	15 (4.6)	13 (8.2)	2 (1.2)	
Once every 2–3 days	35 (10.7)	16 (10.1)	19 (11.4)	
Once every 4–5 days	3 (0.9)	1 (0.6)	2 (1.2)	
Once or less a week	3 (0.9)	3 (1.9)	0 (0)	
**Stool color**, ***n*** **(%)**				<0.001
Yellow brown	238 (73.0)	105 (66.1)	133 (79.6)	
Green	8 (2.5)	7 (4.4)	1 (0.6)	
Black	16 (4.9)	14 (8.8)	2 (1.2)	
White	1 (0.3)	1 (0.6)	0 (0)	
No attention	63 (19.3)	32 (20.1)	31 (18.6)	
**The characteristics of stool**, ***n*** **(%)**				<0.001
Soft and shaped	139 (42.6)	52 (32.7)	87 (52.1)	
Thin strip	82 (25.2)	42 (26.4)	40 (23.9)	
Liquid and shapeless	48 (14.7)	31 (19.5)	17 (10.2)	
Dry and hard	12 (3.7)	11 (6.9)	1 (0.6)	
Watery	0 (0)	0 (0)	0 (0)	
Foamy	2 (0.6)	2 (1.3)	0 (0)	
No attention	43 (13.2)	21 (13.2)	22 (13.2)	

*p-value were calculated by the Mann-Whitney U-test for age, BMI, and the chi-square test for other variables*.

*NA, not applicable*.

According to this investigation, 85.5% of patients with psoriasis vs. 58.1% of healthy controls presented with at least one gastrointestinal symptom. The prevalence of the symptoms, including abdominal pain, abdominal flatulence, borborygmus, gastric acid reflux, belching, nausea or vomiting, passing flatus, urgency of defecation, and constipation, was significantly higher in patients with psoriasis, compared with general population (*p* < 0.01, Table 1). There were significantly different proportion of patients and controls with abnormal stool frequency (20.8 vs. 13.8%), stool color (13.8 vs. 1.8%), and characteristics of stool (54.1 vs. 34.7%).

Results of logistic regression analysis showed that passing flatus and constipation were significantly correlated with psoriasis (*p* < 0.05, respectively, Table 2). Patients with psoriasis were more likely to experience these discomfort symptoms, which suggested possible involvement of gastrointestinal system in psoriasis development.

**Table 2:** Results of logistic regression analysis of gastrointestinal symptoms in psoriasis.

**Variables**	**Odds ratio**	***P***
**Age**	0.97 (0.94~1.00)	0.069
**Sex**	0.69 (0.37~1.31)	0.258
**BMI**	1.13 (1.03~1.23)	0.007
**Abdominal pain**		0.703
No	0.58 (0.23~1.43)	
Slightly	NA	
Moderately	NA	
**Abdominal flatulence**		0.549
No	1.47 (0.71~3.05)	
Slightly	0.69 (0.04~13.16)	
**Borborygmus**		0.992
No	1.16 (0.46~2.91)	
Slightly	NA	
Moderately	NA	
**Gastric acid reflux**		0.832
No	1.47 (0.66~3.28)	
Slightly	NA	
Moderately	NA	
**Backache**		0.468
No	1.65 (0.74~3.67)	
Slightly	0.95 (0.12~7.81)	
**Belching**		0.990
No	0.88 (0.42~1.83)	
Slightly	NA	
Moderately	NA	
**Nausea and vomiting**		0.924
No	1.34 (0.58~3.10)	
Slightly	NA	
Moderately	NA	
**Passing flatus**		0.016
No	3.15 (1.56~6.34)	
Slightly	NA	
Moderately	NA	
**Urgency of defecation**		0.759
No	0.65 (0.25~1.67)	
Slightly	1.69 (0.13~22.17)	
Moderately	NA	
**Constipation**		0.013
No	3.44 (1.58~7.51)	
Slightly	16.20 (0.84~314.55)	
Moderately	NA	
**Stool frequency**		0.330
Once a day	0.99 (0.47~2.07)	
2–3 times a day	4.71 (0.57~39.18)	
More than 3 times a day	0.37 (0.12~1.18)	
Once every 2–3 days	0.15 (0.01~3.51)	
Once every 4–5 days	NA	
**Stool color**		0.245
Yellow brown	6.38 (0.43~95.42)	
No attention	4.85 (0.77~30.50)	
Green	NA	
Black	1.76 (0.78~3.94)	
**The characteristics of stool**		0.884
Soft and shaped	0.95 (0.45~1.99)	
Thin strip	1.14 (0.48~2.98)	
Liquid and shapeless	4.89 (0.40~59.16)	
Dry and hard	NA	
No attention	1.08 (0.41~2.82)	

*NA, not applicable, due to the insufficient number of cases available for the statistics*.

### Psoriasis Improvement Accompanied With Variation of Gut Microbiota

Gut microbiota in patients with psoriasis and healthy controls has been already demonstrated to be significantly different (14, 18). To further understand the relationship between gut microbiota and the degree of psoriasis activity, we then investigated the variation in bacteria community of patients with psoriasis who were effectively treated by acitretin plus NB-UVB (the improvement of PASI score more than 75%).

**Figure 2:**
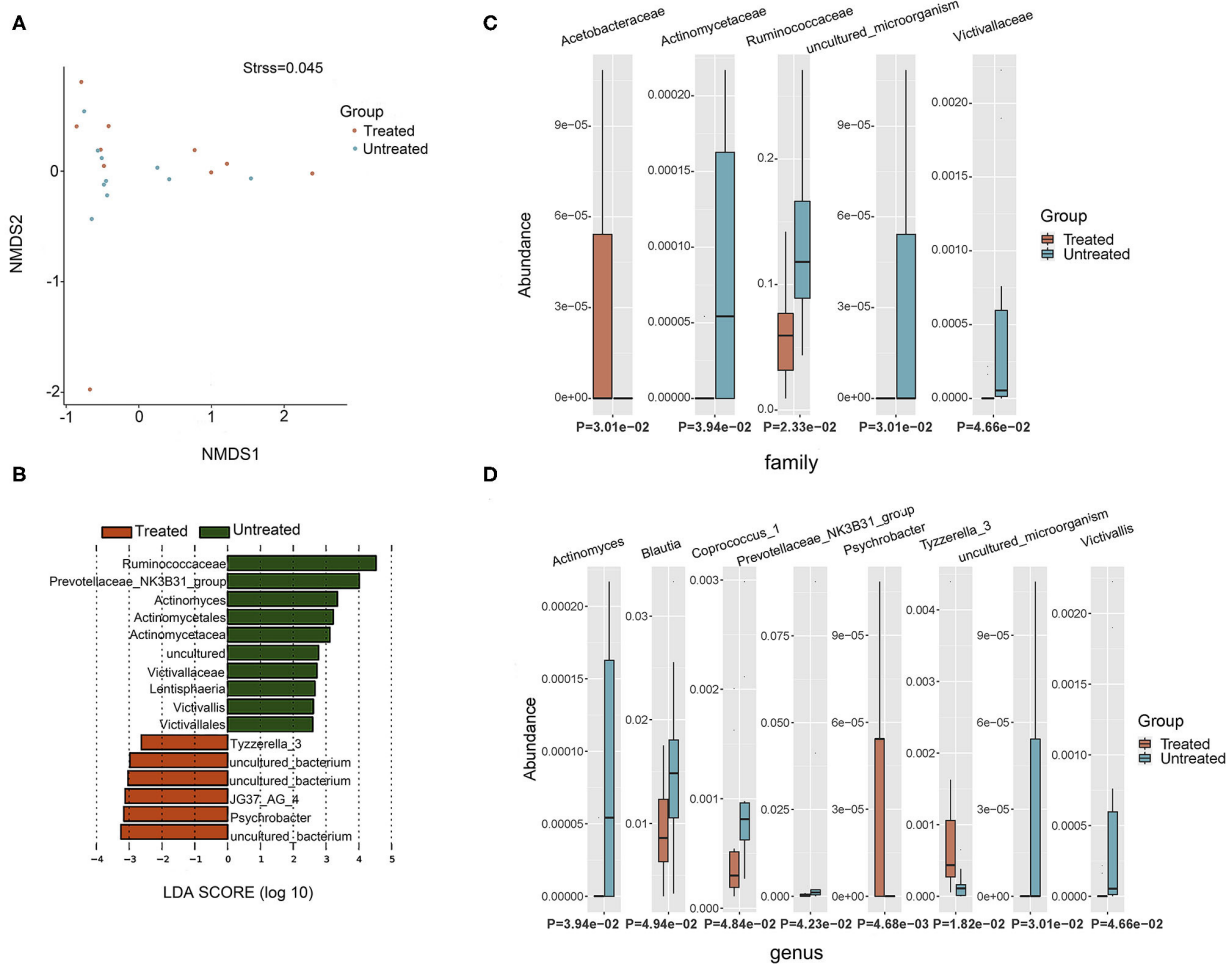
Taxonomic composition of bacterial community in patients with psoriasis (un)treated with acitretin. **(A)** Non-metric Multidimensional Scaling (NMDS) analysis with unweighted UniFrac displayed that most of the untreated group were discriminated from the majority of Treated group samples. Each colored solid circle represents one sample. Solid circles that are closer together represent similar taxonomic composition. **(B)** The scores of linear discriminant analysis for the differentially abundant taxa. Significant bacterial differences at family level **(C)** and at genus level **(D)** between two groups were analyzed by MetaStat analysis.

Two groups of patients (10 untreated and 10 treated with acitretin plus NB-UVB) with moderate-to-severe psoriasis were included (Supplementary Table 1). The gastrointestinal bacterial diversity and composition was evaluated by microbiome analysis based on 16S rRNA. The coverage of applied sequencing depth was adequate, as indicated by goods coverage rarefaction curves of two groups, which tend to be plateau (Supplementary Figure 1A). The results of Alpha diversity indexes indicated similar community richness and species diversity in both groups (*p* = 0.545 for Chao; *p* = 0.112 for Simpson, Supplementary Figures 1B,C). We further applied NMDS to assess the differences of microbial community structure between two groups, and no significant differences were found (Figure 2A, Adonis, *p* = 0.382).

Taxonomic composition revealed differences in the abundance of specific bacterial cluster by LefSe analysis and Kruskal–Wallis analysis (Figures 2B–D). *Bacteroidetes, Firmicutes*, and *Proteobacteria* phyla were dominant in two groups with similar abundance (Supplementary Table 2). This translated to similar *Firmicutes*:*Bacteroides* (*F*/*B*) ratio between two groups (*p* = 0.63), although the median *F*/*B* ratio in Untreated group was higher than that in Treated group (0.637 vs. 0.507). Within identified bacterial components of other taxonomic levels, the comparison between the two groups rendered increased abundance of *JG37_AG_4* class, Acetobacteraceae family, *Psychrobacter* genus and *Tyzzerella_3* genus in Treated group (*p* < 0.05, respectively). Moreover, there were more taxonomical composition of bacteria with decreased relative abundance in Treated group, including Lentisphaeria class, and Victivallales and Actinomycetales at the order level, and Ruminococcaceae, Actinomycetaceae, and Victivallaceae at the family level (*p* < 0.05, respectively). At the genus level, *Actinomyces*, Prevotellaceae _NK3B31_group, *Victivallis, Coprococcus_1*, and *Blautia* were also decreased in Treated group (*p* < 0.05, respectively).

### Delayed Recovery of Psoriasiform Dermatitis in Mice Receiving Psoriatic Microbiota Transplantation

We first confirmed the successful transplantation of human fecal microbiota into mouse psoriasiform models by analyzing the inner structure of mouse microbial community at different time-point (pre-FMT, at 0 and 4th day after complete FMT procedure). Chao1 and Shannon indexes of all samples (five mice per group) were calculated (Supplementary Table 3). Although pre-FMT group and each group at day 0 after FMT (FMT-0d) displayed similar Chao1 index (Supplementary Figures 2A–C), microbial diversity in mice receiving psoriatic fecal microbiota transplantation (PFM-0d group) or mice receiving healthy fecal microbiota transplantation (NFM-0d group) was decreased respectively, compared with pre-FMT group, as determined by Shannon index (*p* < 0.01 for PFM-0d vs. pre-FMT and for NFM-0d vs. pre-FMT, Supplementary Figures 2D–F). The results of PCoA based on unweighted UniFrac distance revealed distinct clustering of seven groups from each other (Supplementary Figures 2G–K). Notably, adonis analyses further revealed that overall microbiota structure differed among seven groups (*p* < 0.05, Supplementary Table 4). These data confirmed that human fecal microbiota was transplanted into mouse psoriasiform models successfully. For recipient mice at all time-point (pre-FMT, at 0 and 4th day after complete FMT procedure), the relative abundance of top 15 bacteria at phylum level was reported in Supplementary Figure 2L.

Imiquimod cream applied onto the shaved back skin of mice can induce skin inflammation accompanied by human psoriasis-like pathological features, including remarkable acanthosis, parakeratosis, and infiltration of inflammatory cells in the superficial dermis. After termination of 1-week IMQ application, typical psoriasis-like phenotypes resolve over time. Here, we investigated the effects of different FMT (from patients with psoriasis or healthy people) on the course of IMQ-induced mouse psoriasiform dermatitis.

**Figure 3:**
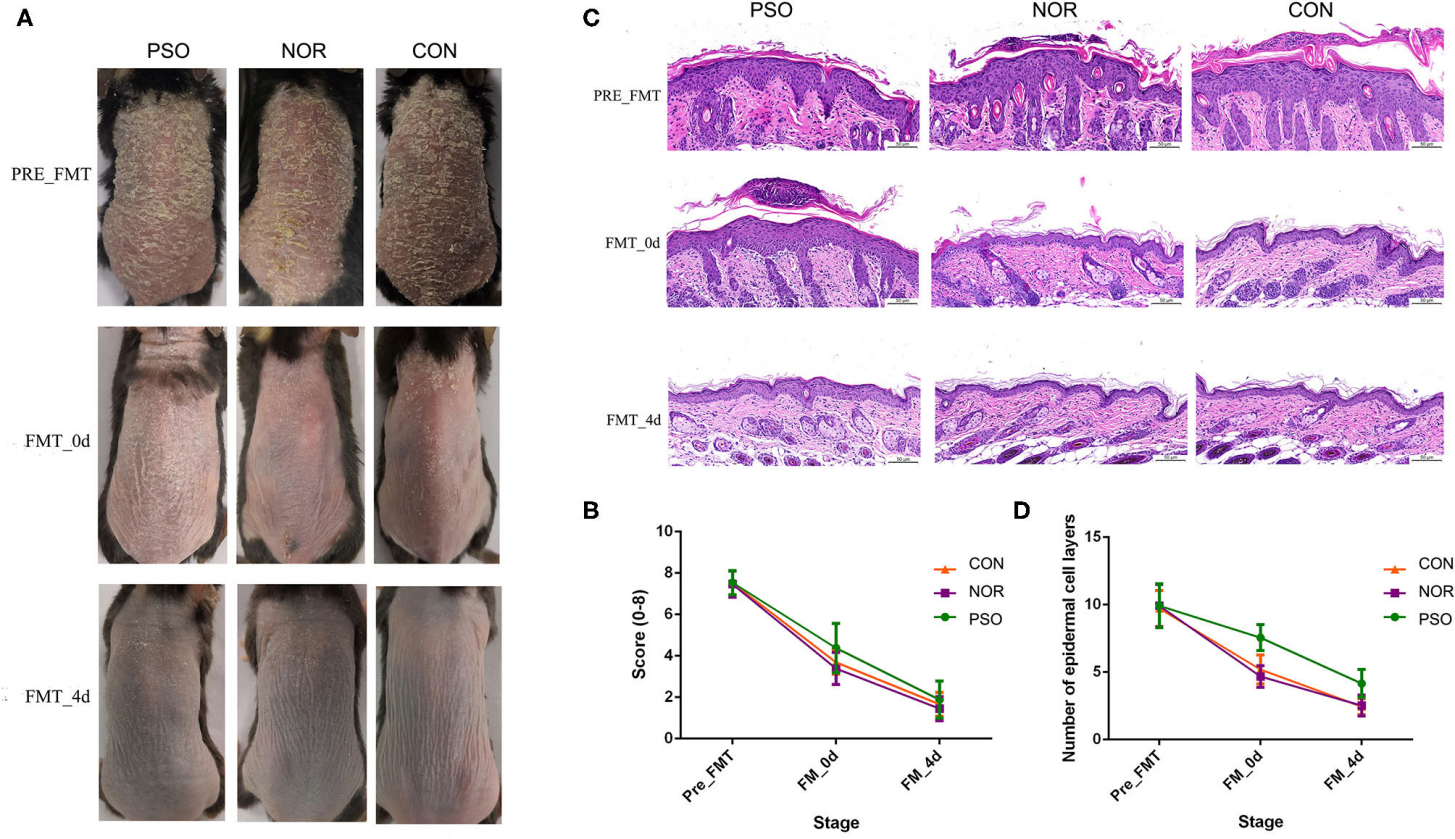
Mice received fecal microbiota transplantation (FMT) from patients with psoriasis showed significantly delayed recovery of psoriasiform dermatitis. After daily application of imiquimod (IMQ) cream for 5 consecutive days, mice in the different groups were respectively transplanted with fecal microbiota from patients with psoriasis (PSO), healthy controls (NOR), or control of PBS (CON). **(A)** Phenotypic presentation of dorsal skin of mice in different group. **(B)** Scores of skin lesions were calculated by erythema plus scaling (a scale from zero to four, respectively). **(C)** H&E staining (scale bar 50 μm) of dorsal skin of mice from different group. **(D)** Epidermal thickness was indicated by number of epidermal cell layers. Colored symbols in **(C,D)** indicated mean score ± SD of five mice per group.

Typical lesions with erythema, scaling, and thickening were observed after 1-week IMQ application, compared with normal mouse skin (Figure 3A, pre-FMT). The cumulative score (erythema plus scaling) was detailed in Supplementary Table 5 and depicted in Figure 3B. At day 0 after 3 consecutive days of FMT, PFM-0d group had higher scores than NFM-0d group or mice receiving PBS (CON-0d group) (*p* < 0.05), and a higher severity of psoriasis-like clinical characteristics was observed. The psoriatic manifestations in all groups almost disappeared at day 4 after FMT, when PFM-4d group, NFM-4d group, and CON-4d group showed similar severity.

Analysis of corresponding pathological slices from IMQ-induced psoriasiform dermatitis revealed relatively thicker epidermis in the PFM group, compared with the NFM group or CON group (*p* < 0.01, Supplementary Table 6, Figures 3C,D). Although there is no significant difference between the two groups, the NFM group presented slightly reduced thickness of the epidermis than the CON group. Thus, mice receiving FMT from patients with psoriasis displayed more delayed recovery of psoriasis-like phenotype up to the end of the experiment.

### Less Reduction of IL-17A in Mice Transplanted With Psoriatic Fecal Microbiota

**Figure 4:**
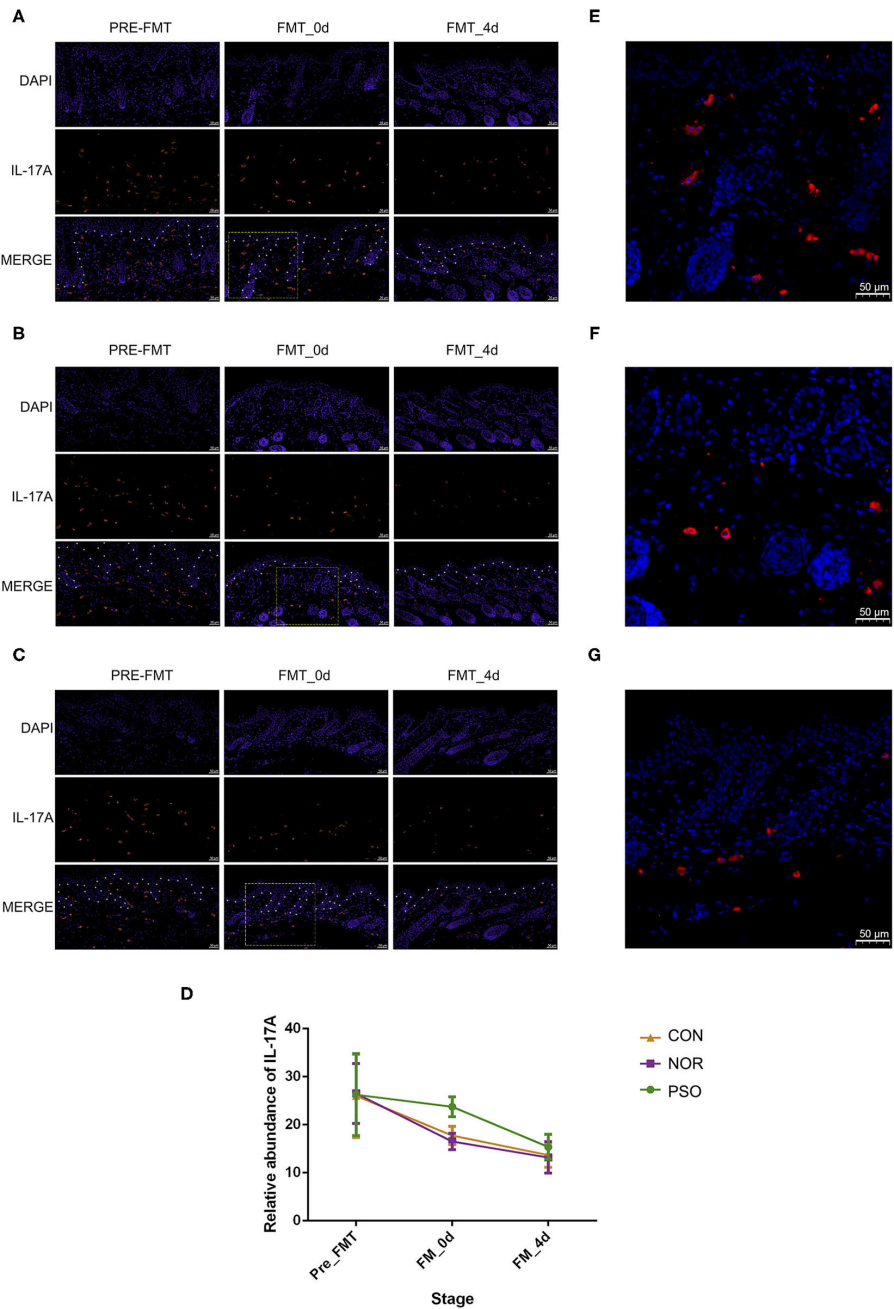
Analysis of IL-17A in mouse skin lesions of psoriasiform by immunofluorescence assay (scale bar 50 μm). **(A)** Mice received FMT of psoriatic fecal sample. **(B)** Mice received FMT of healthy fecal sample. **(C)** Control mice received oral gavage of PBS. Blue fluorescence represents DAPI; red fluorescence represents IL-17A. The borderline between epidermis and dermis was dotted using asterisks. **(D)** Numbers of red fluorescence were counted to analyze IL-17A expression. Colored symbols indicate mean number ± SD of five mice per group. **(E–G)** Are respectively enlarged local area of **(A–C)** at FMT_0d. DAPI, 4′,6-diamidino-2-phenylindole; FMT, fecal microbiota transplantation; PBS, phosphate-buffered saline.

To further verify the possible mechanism of gut microbiota on distal skin changes, we analyzed expression levels of cytokines, including IL-17A, IL-17F, IFN-γ, TNF-α, and IL-23 in psoriasiform mice. The immunofluorescence results showed that IL-17A expression in mouse skin gradually decreased after the termination of IMQ application, accompanied by gradually improved psoriasiform skin lesions (Supplementary Table 7, Figure 4). The level of IL-17A in the PFM-0d group was higher than that in NFM-0d group or CON-0d group, suggesting its less reduction (*p* < 0.01). Although the expression of IL-17A did not differ significantly among three groups at day 4 after FMT, its expression was relatively high in PFM-4d group.

In order to explore the mechanism underlying the less reduction of IL-17A in lesions and considering the possible role of gut–skin axis, we analyzed the changes of IL-17A in gastrointestinal tissues. There was relatively low level of IL-17A expression in gastrointestinal tissues of psoriasiform mice before FMT, and we showed that FMT had an effect on its level (Supplementary Table 8, Supplementary Figure 3). In N and C group, IL-17A increased slightly at day 0 after FMT, and its expression at day 4 after FMT was almost the same as that at pre-FMT (Supplementary Figure 3D). No significant difference between N and C group was observed in all time-points (*p* > 0.05). It should be noted that IL-17A expression in P group was increased in post-FMT (*p* < 0.01), compared with that in N or C group (Supplementary Figure 3). This increase corresponds to the less reduction of IL-17A in lesions. There was no such a corresponding relationship for other cytokines of IL-17F, IL-23, IFN-γ, and TNF-α (data not shown).

## Discussion

Evidence associating gut bacteria with distant extra-intestinal inflammation (e.g., the skin) through regulation of immune system has been expanding (29–31). The clinical observations on psoriatic comorbidities (e.g., IBD) and alterations in architecture of intestinal barrier have fueled the study concerning the correlation between psoriasis pathogenesis/development and gut microbiota.

The present study preliminarily demonstrated the relevance of gut microbiota in the course of psoriasis from several aspects. We first found that multiple gastrointestinal symptoms were significantly more frequent in patients with psoriasis than healthy controls, which may result from compromised intestinal barrier and/or disordered gut microbes (32). Except for gastric acid reflux, other gastrointestinal symptoms involved in this study were not found to be related to gender. Therefore, gender imbalance has little effect on the difference in gastrointestinal symptoms between the two groups.

Later, we found the recovery process of patients with psoriasis was accompanied by reduction of certain “psoriatic characteristic bacterial genera” (13, 15, 16, 33), which had been identified by published studies comparing gut bacteria in patients with psoriasis and healthy controls. Specific bacterial differences at all levels of taxonomic classification were summarized in Supplementary Table 9 (13–18, 33–39). The *F*/*B* ratio has been shown to be significantly related to certain psoriasis comorbidities (e.g., metabolic syndrome) and be positively correlated with PASI score by comparison of gut metagenome between patients with psoriasis and healthy people (15, 37). There was no significant difference of *F*/*B* ratio between patients in Untreated and Treated group. However, it is in line with Dei-Cas et al., where patients with mild psoriasis and patients with moderate-to-severe psoriasis have similar *F*/*B* ratio (38). Specifically, significantly decreased Ruminococcaceae family, *Coprococcus_1* genus, and *Blautia* genus in Treated group corresponds to their significantly increased relative abundance in psoriasis group compared with healthy people. Of them, *Coprococcus_1* and *Blautia* belong to Lachnospiraceae family. However, no difference in relative abundance of the family was found between the two groups. *Blautia* genus is known to be associated with anti-inflammatory properties in allogeneic blood/marrow transplantation, colorectal cancer, and liver cirrhosis (38, 40–43), whereas, *Coprococcus_1* genus and Ruminococcaceae family both are related to antibiotic biosynthesis and have a beneficial influence on gut barrier function by producing short-chain fatty acids (SCFAs) (39, 44, 45). The former is a producer of butyrate, and the latter helps to metabolize indigestible polysaccharides into SCFA (46). Increased SCFAs lead to generate more energy, potentially promoting higher BMI for patients with psoriasis. Moreover, the results of two studies involving gut microbes of patients with psoriasis with different severity are heterogeneous regarding the differences in relative abundance of genus *Bacteroides* among groups. Huang et al. found increased *Bacteroides* in patients with psoriasis, especially the subgroup with severe psoriasis. Our results were consistent with Dei-Cas et al. that no differences of *Bacteroides* were found among patients with different psoriasis status. It is possible that these distinctions were derived from different criteria for the severity of psoriasis (18, 38). There are two hypotheses about the significant reduction of anti-inflammatory bacteria in Treated group and healthy controls. On the one hand, microbiota dysbiosis is one of the trigger factors of psoriasis in genetically predisposed individuals. A mild inflammation caused by other factors induce microbiota to change its composition for immune response, resulting in an abnormal alteration of the immune system. On the other hand, bacterial composition is changed for adjusting immune disorder of psoriasis. Detailed mechanistic studies focused on the causative effect of gut microbiota in psoriasis are required through animal models. At a minimum, these results suggested that gut microbiota was associated with psoriasis development. Unfortunately, there is a lack of patients who did not respond to acitretin plus NB-UVB. This alteration of characteristic microbiota signature might be attributed to acitretin itself or be associated with the recovery of psoriasis directly. However, only one study involved acitretin and gut microbes. It has shown that acitretin inhibits the growth of *Pseudomonas aeruginosa in vitro* (47), which was not a common bacterium in the human intestine. Biological agents were not used in the current study due to their known impact on the composition of the gut microbiome (48, 49). Thus, mechanistic studies with a larger sample size are needed to further explore whether acitretin has an impact on microbes.

We last showed delayed recovery of psoriasiform dermatitis in mice receiving psoriatic microbe transplantation, compared with those receiving healthy microbes. These results further showed a close association between gut microbiota and distal skin inflammation indicating that changes in microbial communities have an impact on the course of psoriasis. Replacing the disordered microbiota with healthy microbes may be beneficial to the recovery of patients with psoriasis. This study is just a preliminary exploration of FMT for the treatment of psoriasis, and further studies are needed.

Recipient mice used in this study were C57BL/six mice, which is superior to mouse with other genetic background, in terms of bacterial diversity and similarity with the donor microbes (26). They were not germ-free or treated with antibiotics like previous literatures reported. Although avoiding interference of their own intestinal microbiota, germ-free and antibiotic-treated mice have some limitations for this study. Antibiotics could not only affect systemic immunity but also limit the colonization of donor microbiota. Similarly, germ-free mice with abnormal intestinal structure have been reported to secrete more lipids resulting in more susceptible to low-grade inflammation or even imbalanced cytokines and immune cells (50). These may be the reason for the fact that germ-free mice and antibiotic-treated mice were more resistant to psoriasis-like inflammation induced by imiquimod, including a lower degree of Th17 activation, less IL-17 production and a lower number of IFN-γ + T cells (51). Antibiotic treatment in mice has been reported to affect the role of *Staphylococcus aureus* and *Streptococcus danieliae* on skin inflammation of imiquimod-induced psoriasis-like dermatitis (52). In addition, richer taxa from human gut have been confirmed to be reliably colonized in mice originally harboring a less diverse microbiota (53). Thus, it is realizable for the FMT in the current study to repopulate mice with microbial community of human.

It is well-accepted that cytokine-mediated inflammatory immune responses are involved in the development of psoriasis, including IL-17, IL-23, TNF-α, etc. (5, 37). *Coprococcus*, which was reduced in Treated group (*p* < 0.05), has been shown to have a strong relationship with abnormal inflammation-related indicators of psoriasis (36). Regarding the related cytokines in this study, the expression of IL-17A in the gastrointestinal tissues of the N and C groups increased slightly on FMT-0d and fell back to that level of pre-FMT on FMT-4d. One likely explanation for the transient rise of IL-17A is mechanical irritation caused by the gavage operation, and the gut microbes from healthy people did not produce obvious immune stimulation to mice. Notably, there was increased IL-17A expression in the gastrointestinal tract of P group post-FMT (*p* < 0.01), which remained higher than that level of pre-FMT, indicating that the disordered microbial communities of patients with psoriasis sustainably affect the immunity of mice. In terms of mechanism, mice in P group with increased IL-17A expression in the gastrointestinal tissue correspondingly showed delayed reduction of IL-17A in lesions and delayed recovery of psoriasiform dermatitis. We speculate the increased IL-17A in the gastrointestinal tract may be the cause of the less reduction of IL-17A in the skin lesion, which in susceptible individuals may trigger the development of psoriasis states. The effect of gut microbiota on the distal skin inflammation seems to be achieved by changing the secretion of cytokines, especially IL-17A, to induce systemic inflammation over-activation in psoriasis. These suggest systemic Th17 over-activation or systemic over-secreted IL-17A circulation may be a link between disordered gut microbiota and psoriasis development. However, the expression of IL-17A in skin lesions of the P group was higher than in N and C groups on FMT-4d, with no statistical difference, accompanied by the recovery of the psoriasis-like phenotype. Given that mice will not develop psoriatic dermatitis in the absence of external factors, the similar IL-17A expression levels among the three groups may be related to the lack of complete psoriasis-related immune pathways in mice. Moreover, the expression levels of other cytokines, including IL-17F, IL-23, IFN-γ, and TNF-α, were of no corresponding relationship with the states of mouse psoriasis-like phenotype. It is plausible that gut microbes may not be involved in different pathways related to these cytokines in mice. Considering the relatively small sample size of our verification analysis, the analysis of interaction between psoriatic course and gut microbiome was likely underpowered and should be further confirmed by studies with larger sample size.

These investigations, observations, and previous published data suggest manipulation of gut microbiota could be a complementary treatment for psoriasis. It was also confirmed by diet adjustment and intake of the probiotic mixture (54, 55). But the efficacy still has some limitation. It is necessary to explore other ways of manipulating the microbiota, such as healthy microbe transplantation. There is a preliminary exploration that found significant clinical efficacy and safety of FMT in the treatment of a patient with severe psoriasis combined with irritable bowel syndrome (56). It is worth noting that the manipulation should aim to targeting the whole community rather than focusing on certain taxa, considering the unknown complex interactions among bacterial microorganisms. However, there are some limitations on using stool samples. Considering that certain microorganisms would loss viability when they are separated from the human body, FMT with fecal samples cannot accurately simulate the state of the bacteria in the donor gut. In addition, stool samples increased the contact between bacteria in different places of the gut, without involving the changes in distribution of microbes along the intestine and differentiating mucosal- and luminal-associated microbiota. Therefore, further exploration on how to make gut microorganisms of the recipient completely equal with their state in the donor body is clearly warranted.

In conclusion, multiple evidence we provided here further supports the involvement of gut microbiota in psoriatic development. This knowledge provides conceivable promise for developing beneficial complementary therapeutics for chronic course management of psoriasis. However, further explorations and clinical trials are needed to confirm the validity and safety of FMT in psoriasis.

## Data Availability Statement

The datasets presented in this study can be found in online repositories. The names of the repository/repositories and accession number(s) can be found at: https://www.ncbi.nlm.nih.gov/, PRJNA743747; https://www.ncbi.nlm.nih.gov/, PRJNA741854.

## Ethics Statement

The studies involving human participants were reviewed and approved by the Ethics Committee of Sichuan Provincial People's Hospital. The patients/participants provided their written informed consent to participate in this study. The animal study was reviewed and approved by the Ethics Committee of Sichuan Provincial People's Hospital.

## Author Contributions

CS contributed to literature search, operation in experiments, data analysis, statistical analysis, and drafting of the manuscript. LC contributed to conception of the study, data collection/analysis, literature search, and critical revision of the manuscript. HY contributed to statistical analysis. HS contributed to operation in experiments and statistical analysis. ZX contributed to data interpretation and literature search. BZ contributed to data collection/analysis. XJ contributed to sample collection and data collection. BQ contributed to operation in experiments. ZS contributed to conception and design of the study, literature search, data collection, statistical analysis, and critical revision of the manuscript. All authors have read and approved the final manuscript.

## Funding

This work was supported by National Natural Science Foundation of China (Nos. 81771783 and 82073444).

## Conflict of Interest

The authors declare that the research was conducted in the absence of any commercial or financial relationships that could be construed as a potential conflict of interest.

## Publisher's Note

All claims expressed in this article are solely those of the authors and do not necessarily represent those of their affiliated organizations, or those of the publisher, the editors and the reviewers. Any product that may be evaluated in this article, or claim that may be made by its manufacturer, is not guaranteed or endorsed by the publisher.

## Supplementary Material

Supplementary Figure 1 Similar community richness and species diversity in patients with psoriasis (un)treated with acitretin. **(A)** Obtained goods coverage index rarefaction curves all tended to be plateau, as the reading increases. Box plots with **(B)** Chao1 and **(C)** Simpson indexes were depicted.

Supplementary Figure 2 Bacterial Alpha diversity and Beta diversity in all groups of mice. Chao1 index **(A)** among pre-FMT, PFM-0d, and PFM-4d, **(B)** among pre-FMT, NFM-0d, and NFM-4d, and **(C)** among pre-FMT, CON-0d, and CON-4d were depicted with Box plots and analyzed by Wilcoxon rank-sum test. Shannon index **(D)** among pre-FMT, PFM-0d, and PFM-4d, **(E)** among pre-FMT, NFM-0d, and NFM-4d, and **(F)** among pre-FMT, CON-0d, and CON-4d were depicted with Box plots and analyzed by Wilcoxon rank-sum test. ^*^*p* < 0.05 and ^**^*p* < 0.01. Principal coordinate analysis (PCoA) based on unweighted UniFrac distance showed differential clustering **(G)** among pre-FMT, PFM-0d, and PFM-4d, **(H)** among pre-FMT, NFM-0d, and NFM-4d, **(I)** among pre-FMT, CON-0d, and CON-4d, **(J)** among pre-FMT, PFM-0d, NFM-0d, and CON-0d, and **(K)** among pre-FMT, PFM-4d, NFM-4d, and CON-4d. Solid circles in the same circle represent similar bacterial composition. PC1, principal coordinate 1; PC2, principal coordinate 2. The percentage indicates the contribution of each principal component to the difference. **(L)** The relative abundance of top 15 bacteria at phylum level of all groups. Pre-FMT, before FMT; PFM-0d, at day 0 after FMT of psoriatic fecal sample; NFM-0d, at day 0 after FMT of normal fecal sample; CON-0d, at day 0 after oral gavage of PBS; PFM-4d, at day 4 after FMT of psoriatic fecal sample; NFM-4d, at day 4 after FMT of normal fecal sample; CON-4d, at day 4 after oral gavage of PBS.

Supplementary Figure 3 Analysis of IL-17A in mouse gastrointestinal tissues of psoriasiform models by immunofluorescence assay (scale bar 50 μm). **(A)** Mice received FMT of psoriatic fecal sample. **(B)** Mice received FMT of healthy fecal sample. **(C)** Control mice received oral gavage of PBS. Blue fluorescence represents DAPI; red fluorescence represents IL-17A. **(D)** Numbers of red fluorescence were counted to analyze IL-17A expression. Colored symbols indicate mean number ± SD of five mice per group. **(E–G)** Are respectively enlarged local area of **(A–C)** at FMT_0d.

Supplementary Appendix 1 Main investigation content of gastrointestinal discomfort symptoms in psoriatic patients and general population.

Supplementary Table 1 The demographic details of psoriatic patients (un)treated with Acitretin plus NB-UVB. PASI 75, clinical improvement more than 75%, assessed by PASI score.

Supplementary Table 2 The relative abundance and difference of each identified phylum between Untreated group and Treated group. *P*-value was calculated by kruskal-wallis analysis. FDR_P, *P*-value calibrated by False Discovery Rate; Untreated_mean, mean relative abundance of each bacterial phylum in Untreated group; Treated_mean, mean relative abundance of each bacterial phylum in Treated group.

Supplementary Table 3 The chao1 and shannon index of mouse fecal microbiota samples. FMT, fecal microbiota transfer; Pre-FMT, before FMT; PFM-0d, at day 0 after FMT of psoriatic fecal sample; NFM-0d, at day 0 after FMT of healthy fecal sample; CON-0d, at day 0 after oral gavage of PBS; PFM-4d, at day 4 after FMT of psoriatic fecal sample; NFM-4d, at day 4 after FMT of healthy fecal sample; CON-4d, at day 4 after oral gavage of PBS.

Supplementary Table 4 The results of Adonis analysis among groups. FMT, fecal microbiota transfer; Pre-FMT, before FMT; PFM-0d, at day 0 after FMT of psoriatic fecal sample; NFM-0d, at day 0 after FMT of healthy fecal sample; CON-0d, at day 0 after oral gavage of PBS; PFM-4d, at day 4 after FMT of psoriatic fecal sample; NFM-4d, at day 4 after FMT of healthy fecal sample; CON-4d, at day 4 after oral gavage of PBS.

Supplementary Table 5 The scores of the skin scaling and erythema in mouse psoriasiform models measured by four independent researchers. FMT, fecal microbiota transplantation; FMT-0d, Day 0 after FMT; FMT-4d, Day 4 after FMT; P group, mice receiving psoriatic microbiota transplantation; N group, mice receiving healthy microbiota transplantation; C group, mice receiving PBS; Mean (SD), Mean and SD of PASI scores (scales plus erythema).

Supplementary Table 6 The number of epidermal cell layers in the back skin of mice psoriasiform models measured by three independent researchers under two high-power fields of light microscope. FMT, fecal microbiota transplantation; FMT-0d, Day 0 after FMT; FMT-4d, Day 4 after FMT; P group, mice receiving psoriatic microbiota transplantation; N group, mice receiving healthy microbiota transplantation; C group, mice receiving PBS; Mean (SD), Mean and SD of number of epidermal cell layers under one high-power fields.

Supplementary Table 7 The number of IL-17A^+^ cells in lesion of mouse psoriasiform models. PFM, mice receiving psoriatic fecal microbiota transplantation; NFM, mice receiving healthy fecal microbiota transplantation; CON, mice receiving PBS; PRE, Pre-fecal-microbiota-transplantation; 0d, at Day 0 after fecal microbiota transplantation; 4d, at Day 4 after fecal microbiota transplantation; M n, n mice in the same group.

Supplementary Table 8 The number of IL-17A^+^ cells in gastrointestinal tissues of mouse psoriasiform models. PFM, mice receiving psoriatic fecal microbiota transplantation; NFM, mice receiving healthy fecal microbiota transplantation; CON, mice receiving PBS; PRE, Pre-fecal-microbiota-transplantation; 0d, at Day 0 after fecal microbiota transplantation; 4d, at Day 4 after fecal microbiota transplantation; M n, n mice in the same group.

Supplementary Table 9 Gut microbial alterations in patients with psoriasis/psoriatic arthritis compared with normal controls, based on published literatures.
