# Supplementary material for: Involvement of Gut Microbiota in the Development of Psoriasis Vulgaris
Source: Front Nutr. 2021 Nov 22;8:761978. doi: 10.3389/fnut.2021.761978 (PMC8646027; doi:10.3389/fnut.2021.761978)
Supplement: Supplementary Figure 1 — Similar community richness and species diversity in patients with psoriasis (un)treated with acitretin. (A) Obtained goods coverage index rarefaction curves all tended to be plateau, as the reading increases. Box plots with (B) Chao1 and (C) Simpson indexes were depicted. [file Data_Sheet_1.doc]

**Supplementary Fig S1**


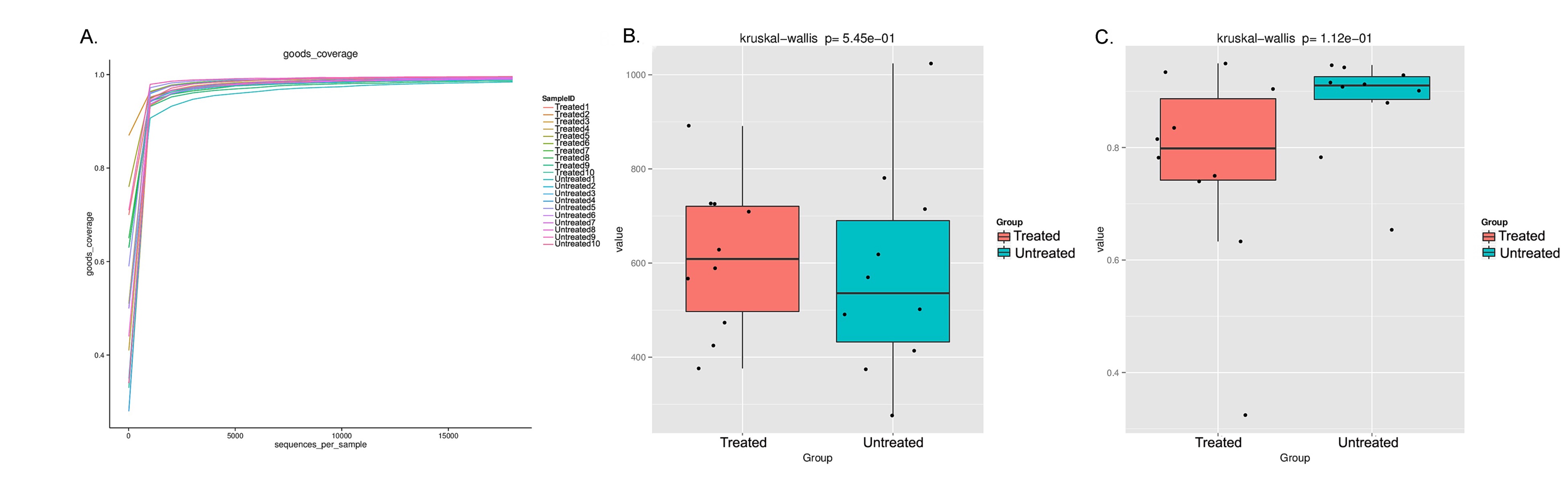


**Supplementary Fig S2**


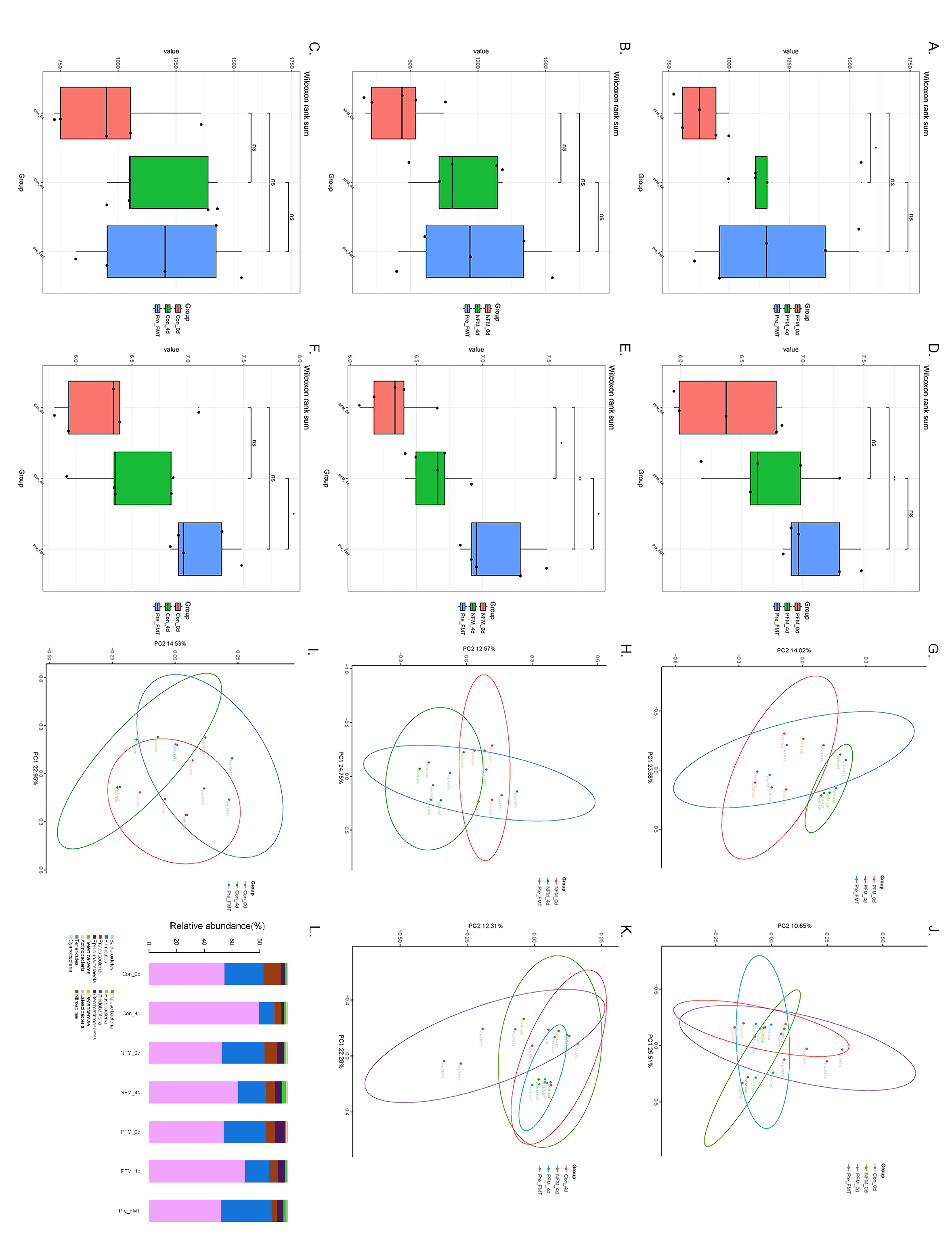


**Supplementary Fig S3**

**
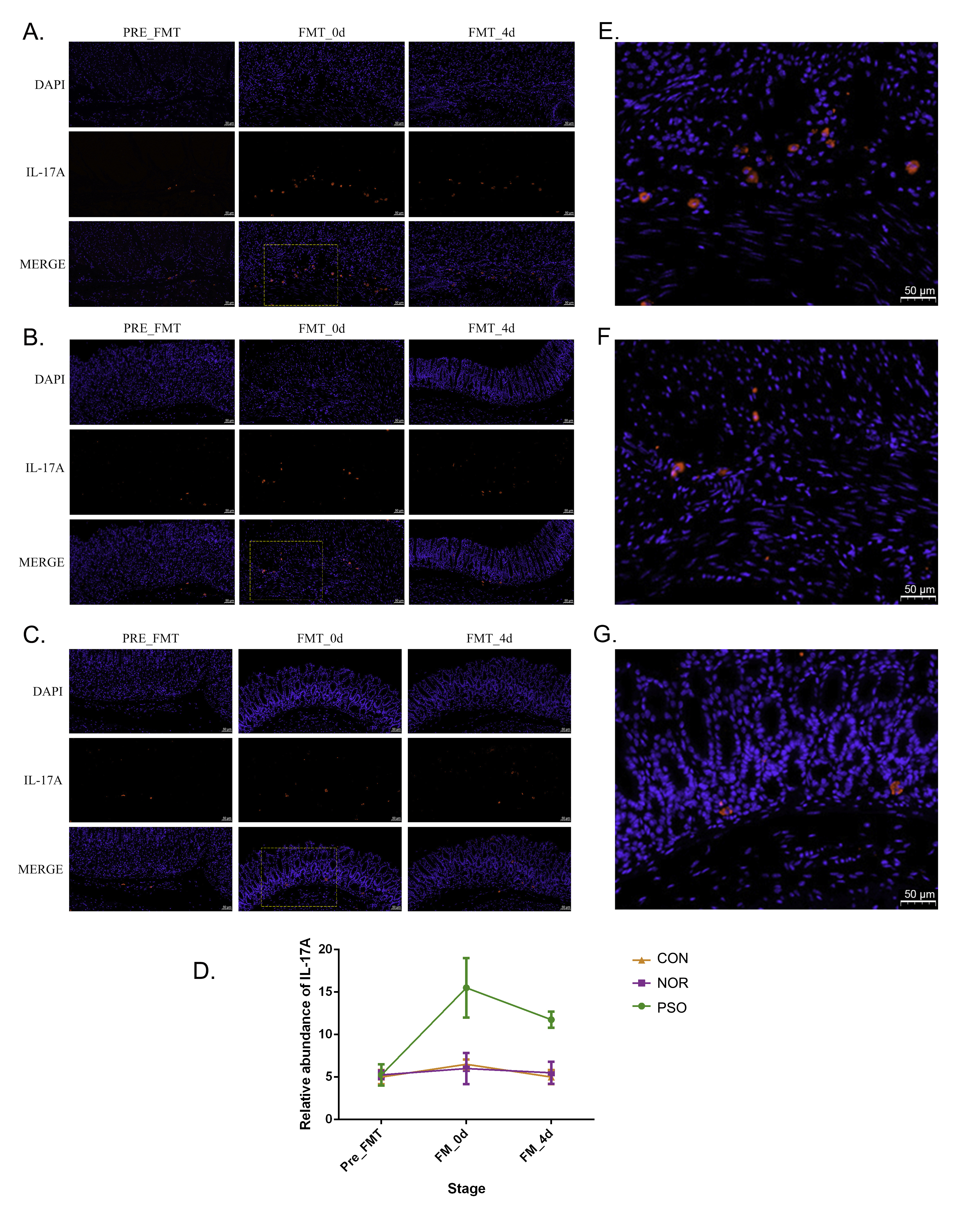
**

**Appendix S1. Main investigation content of gastrointestinal discomfort symptoms in psoriatic patients and general population.**

**1. In the past five years, your physical condition:**

① No psoriasis;

② Psoriasis diagnosed by at least one dermatologist

If ① chosen, go directly to **5**.
**2. The total course of your psoriasis:**

① < 3 months;

② ≥ 3, and < 6 months;

③ ≥ 6, and < 12 months;

④ ≥ 1, and < 3 years;

⑤ ≥ 3, and < 5 years;

⑥ ≥ 5 years

**3. Severity of your psoriasis in the last five years:**

① Not affecting daily life at all;

② Slightly affecting daily life;

③ Seriously affecting daily life

**4. Mean area of your psoriasis lesions in the last five years:**

① < 1 palm;

② ≥ 1, and < 5 palms;

③ ≥ 5, and < 10 palms;

④ ≥ 10 palms

**5. Full name**： （Optional）

**6. Tel**： （Optional）

**7. Gender**：

① male;

② female

**8. Date of birth**： year month

**9. Nationality**：

① Han nationality;

② Other

**10. Marriage status**：

① unmarried;

② married;

③ divorced;

④ widowed

**11. Weight (kg)**：

**12. Height (CM)**：

**13. Long-term residence in recent five years**： （province）

**14. Education background：**

① primary school;

② junior high school;

③ senior high school or technical secondary school;

④ university or junior college;

⑤ postgraduate

**15. Monthly income (Yuan)**:

① none;

② < 3000;

③ ≥ 3000, and < 5000;

④ ≥ 5000, and < 10000;

⑤ ≥ 10000

**Do you have the following symptoms:**

**16. Abdominal pain**:

① no or transient pain;

② occasional pain affecting part of social activities;

③ prolonged pain affecting most social activities and requiring treatments;

④ severe pain affecting all social activities

**17. Types of abdominal pain**:

① colic pain;

② dull pain;

③ tingling pain;

④ cold pain;

⑤ distending pain;

⑥ other pain;

⑦ no pain

**18. Abdominal flatulence**:

① no or transient abdominal flatulence;

② occasional abdominal flatulence;

③ frequent and long-term abdominal flatulence;

④ continuous abdominal flatulence, which seriously affects social activities

**19. Borborygmus:**

① no or transient barborygmus;

② temporary and occasional barborygmus;

③ frequent and long-term barborygmus;

④ continuous barborygmus, which seriously affects social activities

**20. Gastric acid reflux**:

① no or transient reflux;

② occasional reflux;

③ reflux 1-2 times per day, and needs to be treated for relief;

④ reflux several times per day, and anti-acid treatment can only obtain partial relief

**21. Back pain:**

① no or transient pain;

② occasional pain to affect part of social activities;

③ prolonged pain to require treatment, and affecting many social activities;

④ severe pain to affect all social activities

**22. Belching**:

① no or transient belching;

② occasional belching;

③ frequent and long-term belching, and require treatments for control;

④ persistent belching affecting social activities

**23. Nausea or vomiting**:

① no nausea;

② occasionally nausea;

③ frequent and long-term nausea, without vomiting;

④ persistent nausea with vomiting

**24. Passing flatus**:

① no increased flatus;

② temporary or occasional increased flatus;

③ increased flatus affecting partial social activities;

④ increased flatus seriously affecting social activities

**25. Urgency of defecation**:

① normal control;

② occasional sense of urgency in defecation;

③ frequent sense of urgency in defecation, affecting social activities;

④ fecal incontinence

**26. Constipation**:

① no constipation;

② occasional constipation;

③ difficulty in defecation, usually accompanied by feeling of endless defecation;

④ severe constipation, and a treatment is necessary to defecate

**27. Stool frequency**:

① once a day;

② 2-3 times per day;

③ more than 3 times per day;

④ once every 2-3 days;

⑤ once every 4-5 days;

⑥ once a week or less

**28. Stool color**:

① yellow brown;

② green;

③ black;

④ white;

⑤ no attention

**29. The characteristics of stool:**

① soft and shaped;

② thin strip;

③ liquid and shapeless;

④ dry and hard;

⑤ watery;

⑥ foamy;

⑦ no attention

**Supplementary Table S1. The demographic details of psoriatic patients (un)treated with Acitretin** **plus NB-UVB**

|  | **Untreated patients** | **Acitretin-treated, over PASI75** | **p value** |
| --- | --- | --- | --- |
| Sex, n (%) |  |  | 1.0 |
| Male | 5 (50%) | 5 (50%) |  |
| Female | 5 (50%) | 5 (50%) |  |
| Age, mean ± SD | 37.2 ± 14.19 | 36.2 ± 10.58 | 0.87 |
| Disease duration | 4.6 ± 2.37 (year) | 4.78 ± 2.31 (year) | 0.88 |
| PASI score | 15.3 ± 4.11 | 3.6 ± 1.43 | < 0.001 |

PASI 75, clinical improvement more than 75%, assessed by PASI score.

**Supplementary Table S2. The relative abundance and difference of each identified phylum between Untreated group and Treated group**

| **OTU** | **Test-Statistic** | **P** **value** | **FDR_P** | **Untreated_mean** | **Treated_mean** |
| --- | --- | --- | --- | --- | --- |
| Actinobacteria | 2.643416 | 0.10398 | 0.901771 | 0.00922 | 0.003857 |
| Chloroflexi | 2.568276 | 0.109027 | 0.901771 | 0.000239 | 0.00038 |
| TA06 | 2.4025 | 0.121142 | 0.901771 | 5.43E-06 | 2.71E-05 |
| Lentisphaerae | 2.158589 | 0.141775 | 0.901771 | 0.000564 | 7.60E-05 |
| Euryarchaeota | 2.111111 | 0.146233 | 0.901771 | 1.09E-05 | 0 |
| Kazan_3B_09 | 2.111111 | 0.146233 | 0.901771 | 1.09E-05 | 0 |
| Firmicutes | 1.462857 | 0.226476 | 0.973814 | 0.344135 | 0.283062 |
| LCP_89 | 1 | 0.317311 | 0.973814 | 1.09E-05 | 0 |
| Elusimicrobia | 1 | 0.317311 | 0.973814 | 1.09E-05 | 0 |
| JL_ETNP_Z39 | 1 | 0.317311 | 0.973814 | 0 | 5.43E-06 |
| WCHB1_60 | 1 | 0.317311 | 0.973814 | 0 | 5.43E-06 |
| Woesearchaeota_(DHVEG_6) | 1 | 0.317311 | 0.973814 | 0 | 5.43E-06 |
| Verrucomicrobia | 0.902356 | 0.342151 | 0.973814 | 0.000467 | 0.000749 |
| Fusobacteria | 0.632377 | 0.426485 | 0.975313 | 0.00427 | 0.02264 |
| Other | 0.600316 | 0.438458 | 0.975313 | 0.000157 | 0.000222 |
| Cyanobacteria | 0.521695 | 0.47012 | 0.975313 | 0.004655 | 5.43E-05 |
| Candidate_division_OP3 | 0.448148 | 0.503216 | 0.975313 | 5.43E-06 | 2.17E-05 |
| Armatimonadetes | 0.448148 | 0.503216 | 0.975313 | 5.43E-06 | 1.63E-05 |
| Saccharibacteria | 0.372549 | 0.541618 | 0.975313 | 1.09E-05 | 5.43E-06 |
| Hyd24_12 | 0.372549 | 0.541618 | 0.975313 | 1.09E-05 | 5.43E-06 |
| Aminicenantes | 0.350985 | 0.553556 | 0.975313 | 8.14E-05 | 8.14E-05 |
| Acidobacteria | 0.281057 | 0.59601 | 0.99252 | 0.000971 | 0.001042 |
| Bacteroidetes | 0.205714 | 0.650147 | 0.99252 | 0.540506 | 0.558209 |
| Chlorobi | 0.146266 | 0.702129 | 0.99252 | 0.000244 | 0.000288 |
| Spirochaetae | 0.100496 | 0.751235 | 0.99252 | 9.22E-05 | 7.60E-05 |
| Nitrospirae | 0.073077 | 0.786908 | 0.99252 | 0.000195 | 0.000239 |
| Gemmatimonadetes | 0.072905 | 0.787153 | 0.99252 | 0.00013 | 0.000152 |
| Tenericutes | 0.056763 | 0.811687 | 0.99252 | 0.000369 | 0.000195 |
| Latescibacteria | 0.054633 | 0.81519 | 0.99252 | 0.000114 | 0.000125 |
| Proteobacteria | 0.051429 | 0.820596 | 0.99252 | 0.093213 | 0.128163 |
| Synergistetes | 0.032169 | 0.857657 | 0.99252 | 3.26E-05 | 3.26E-05 |
| WD272 | 0.031832 | 0.858396 | 0.99252 | 3.80E-05 | 2.71E-05 |
| Planctomycetes | 0.013287 | 0.908233 | 1 | 0.000168 | 0.00019 |
| Deferribacteres | 0 | 1.0 | 1 | 2.71E-05 | 2.71E-05 |
| Hydrogenedentes | 0 | 1.0 | 1 | 1.09E-05 | 1.09E-05 |
| Chlamydiae | 0 | 1.0 | 1 | 5.43E-06 | 5.43E-06 |
| Thaumarchaeota | 0 | 1.0 | 1 | 5.43E-06 | 5.43E-06 |

P value was calculated by kruskal-wallis analysis. FDR_P, P value calibrated by False Discovery Rate; Untreated_mean, mean relative abundance of each bacterial phylum in Untreated group; Treated_mean, mean relative abundance of each bacterial phylum in Treated group.

**Supplementary Table S3. The chao1 and shannon index of mouse fecal microbiota samples**

| Samples | Chao1 | Shannon |
| --- | --- | --- |
| Con-0d1 | 1427.412366 | 7.083762783 |
| Con-0d2 | 693.9056609 | 5.812114353 |
| Con-0d3 | 745.3677374 | 5.924835869 |
| Con-0d4 | 955.8363097 | 6.334937819 |
| Con-0d5 | 1040.601564 | 6.390935075 |
| Con-4d2 | 1404.350522 | 6.869765482 |
| Con-4d5 | 1394.1839 | 6.845933101 |
| Con-4d6 | 1034.892542 | 6.346849332 |
| Con-4d7 | 983.564959 | 5.90159147 |
| Con-4d8 | 1051.095093 | 6.371023789 |
| NFM-0d1 | 722.8400048 | 6.188334112 |
| NFM-0d2 | 736.1812008 | 6.061082875 |
| NFM-0d3 | 874.331424 | 6.402959453 |
| NFM-0d4 | 932.6558627 | 6.351215467 |
| NFM-0d8 | 1029.130211 | 6.668520123 |
| NFM-4d2 | 894.2941169 | 6.415907515 |
| NFM-4d3 | 1282.112356 | 6.918678015 |
| NFM-4d5 | 1265.67648 | 6.665131929 |
| NFM-4d7 | 1071.451454 | 6.725758603 |
| NFM-4d8 | 1046.178209 | 6.482802508 |
| PFM-0d2 | 989.6800906 | 6.784403473 |
| PFM-0d4 | 964.3946533 | 6.375451488 |
| PFM-0d6 | 861.4572118 | 6.828329634 |
| PFM-0d7 | 785.5969888 | 5.952095405 |
| PFM-0d8 | 826.2216702 | 5.979003807 |
| PFM-4d2 | 1570.220641 | 7.293102382 |
| PFM-4d3 | 1163.058397 | 6.996711093 |
| PFM-4d6 | 1106.731235 | 6.552710418 |
| PFM-4d7 | 1141.645119 | 6.636893968 |
| PFM-4d8 | 976.7248754 | 6.156598005 |
| Pre-FMT1 | 829.5077445 | 6.827606612 |
| Pre-FMT2 | 1149.476838 | 6.962579387 |
| Pre-FMT5 | 1534.09003 | 7.510503819 |
| Pre-FMT6 | 1358.612986 | 7.300778446 |
| Pre-FMT7 | 983.5945057 | 6.912840531 |

FMT, fecal microbiota transfer; Pre-FMT, before FMT; PFM-0d, at day 0 after FMT of psoriatic fecal sample; NFM-0d, at day 0 after FMT of healthy fecal sample; CON-0d, at day 0 after oral gavage of PBS; PFM-4d, at day 4 after FMT of psoriatic fecal sample; NFM-4d, at day 4 after FMT of healthy fecal sample; CON-4d, at day 4 after oral gavage of PBS.

**Supplementary Table S4. The results of Adonis analysis among groups**

|  | **F.Model** | **Pr(>F)** |
| --- | --- | --- |
| Pre-FMT vs PFM-0d vs PFM-4d | 1.6741 | 0.024 |
| Pre-FMT vs NFM-0d vs NFM-4d | 1.4693 | 0.047 |
| Pre-FMT vs PFM-0d vs NFM-0d vs CON-0d vs CON-4d vs NFM-4d vs PFM-4d | 1.6285 | 0.001 |

FMT, fecal microbiota transfer; Pre-FMT, before FMT; PFM-0d, at day 0 after FMT of psoriatic fecal sample; NFM-0d, at day 0 after FMT of healthy fecal sample; CON-0d, at day 0 after oral gavage of PBS; PFM-4d, at day 4 after FMT of psoriatic fecal sample; NFM-4d, at day 4 after FMT of healthy fecal sample; CON-4d, at day 4 after oral gavage of PBS.

**Supplementary Table S5. The scores of the skin scaling and erythema in mouse psoriasiform models measured by four independent researchers.**

|  | scales/erythema (researcher 1) | scales/erythema (researcher 2) | scales/erythema (researcher 3) | scales/erythema (researcher 4) | Mean (SD) |
| --- | --- | --- | --- | --- | --- |
| **Pre-FMT (P group)** |  |  |  |  | 7.535  (0.5833) |
| Mouse 1 | 3.5 / 4 | 3.9 / 4 | 3.6 / 3 | 3.5 / 4 |  |
| Mouse 2 | 4 / 4 | 4 / 3.9 | 3.7 / 2.8 | 4 / 4 |  |
| Mouse 3 | 4 / 4 | 3.9 / 3.9 | 3.7 / 2.5 | 3.5 / 3.5 |  |
| Mouse 4 | 4 / 4 | 4 / 3.9 | 3.8 / 3 | 3.8 / 4 |  |
| Mouse 5 | 4 / 4 | 4 / 4 | 4 / 3.5 | 3.8 / 4 |  |
| **Pre-FMT (N group)** |  |  |  |  | 7.465 (0.6377) |
| Mouse 1 | 4 / 4 | 3.8 / 4 | 3.5 / 3 | 4 / 4 |  |
| Mouse 2 | 3.5 / 4 | 3.8 / 3.9 | 3 / 3.5 | 3.8 / 4 |  |
| Mouse 3 | 3.5 / 4 | 4 / 4 | 3.6 / 2.7 | 3.9 / 3.9 |  |
| Mouse 4 | 4 / 4 | 3.9 / 3.9 | 3.5 / 2.9 | 4 / 3.7 |  |
| Mouse 5 | 4 / 4 | 4 / 3.9 | 3 / 3.5 | 3.9 / 3.8 |  |
| **Pre-FMT (C group)** |  |  |  |  | 7.520 (0.5996) |
| Mouse 1 | 4 / 4 | 3.9 / 3.9 | 3.4 / 3.6 | 3.9 / 4 |  |
| Mouse 2 | 4 / 4 | 4 / 3.9 | 3 / 3.5 | 4 / 4 |  |
| Mouse 3 | 4 / 4 | 4 / 4 | 3 / 3.6 | 3.9 / 3.9 |  |
| Mouse 4 | 4 / 3.5 | 3.9 / 3.9 | 2.9 / 3.8 | 3.7 / 4 |  |
| Mouse 5 | 3.8 / 3.8 | 3.7 / 4 | 3 / 3.1 | 3.8 / 3.6 |  |
| **FMT-0d (P group)** |  |  |  |  | 4.375 (1.184) |
| Mouse 1 | 2.5 / 3 | 2.5 / 2.7 | 2 / 2 | 2 / 4 |  |
| Mouse 2 | 2 / 2 | 2 / 2 | 1.2 / 1.2 | 2 / 3.5 |  |
| Mouse 3 | 2 / 2 | 2.1 / 2 | 1.2 / 1.2 | 1.5 / 3 |  |
| Mouse 4 | 2 / 2 | 2.1 / 2 | 1 / 1 | 2.5 / 3 |  |
| Mouse 5 | 2.5 / 2.5 | 2.5 / 2.3 | 2 / 2 | 3 / 3.5 |  |
| **FMT-0d (N group)** |  |  |  |  | 3.390 (0.7745) |
| Mouse 1 | 2 / 2 | 2 / 2 | 1.4 / 1.1 | 1.5 / 2 |  |
| Mouse 2 | 2 / 2 | 2 / 2 | 1.2 / 1.3 | 1.5 / 2 |  |
| Mouse 3 | 2 / 2 | 2.1 / 1.9 | 1.3 / 1.1 | 2 / 2 |  |
| Mouse 4 | 2 / 2 | 1.8 / 1.6 | 1 / 0.9 | 1 / 1 |  |
| Mouse 5 | 2.5 / 2.5 | 1.7 / 2.4 | 1 / 1.5 | 1.5 / 2 |  |
| **FMT-0d (C group)** |  |  |  |  | 3.685 (0.5650) |
| Mouse 1 | 2 / 2 | 1.9 / 2.2 | 1.5 / 1.4 | 2 / 2 |  |
| Mouse 2 | 2 / 2 | 1.9 / 2 | 1.2 / 1.5 | 1.5 / 1.5 |  |
| Mouse 3 | 2 / 2 | 1.9 / 2 | 1.3 / 1.5 | 2 / 2 |  |
| Mouse 4 | 2.5 / 2 | 2.4 / 1.8 | 1.7 / 1.3 | 2 / 1.5 |  |
| Mouse 5 | 2 / 2 | 2 / 2.5 | 1.5 / 1.7 | 1.5 / 2 |  |
| **FMT-4d (P group)** |  |  |  |  | 1.890 (0.8914) |
| Mouse 1 | 1 / 1.5 | 0.5 / 1.3 | 0.5 / 0.6 | 1.5 / 2 |  |
| Mouse 2 | 1 / 1 | 0.7 / 0.7 | 0.6 / 0.4 | 2 / 1.5 |  |
| Mouse 3 | 1 / 0.5 | 0.8 / 0.7 | 0.8 / 0.4 | 2 / 1.5 |  |
| Mouse 4 | 0.5 / 0.5 | 1 / 0.3 | 0.7 / 0.3 | 1.5 / 1 |  |
| Mouse 5 | 1 / 1 | 0.8 / 0.7 | 0.6 / 0.4 | 1.5 / 1.5 |  |
| **FMT-4d (N group)** |  |  |  |  | 1.455 (0.5835) |
| Mouse 1 | 1 / 1 | 0.8 / 0.7 | 0.9 / 0.3 | 1.5 / 1 |  |
| Mouse 2 | 0.5 / 0.5 | 0.5 / 0.5 | 0.6 / 0.3 | 1 / 1 |  |
| Mouse 3 | 1 / 1 | 0.8 / 0.5 | 0.8 / 0.3 | 2 / 1 |  |
| Mouse 4 | 0.5 / 0.5 | 0.6 / 0.3 | 0.5 / 0.3 | 0.5 / 0.5 |  |
| Mouse 5 | 1 / 0.5 | 1 / 0.4 | 1.2 / 0.3 | 1 / 0.5 |  |
| **FMT-4d (C group)** |  |  |  |  | 1.660 (0.5707) |
| Mouse 1 | 0.5 / 1 | 0.4 / 1 | 0.3 / 0.5 | 1.5 / 1.5 |  |
| Mouse 2 | 1 / 0.5 | 1 / 0.7 | 1 / 0.4 | 1.5 / 1 |  |
| Mouse 3 | 1 / 1 | 0.9 / 1.4 | 0.8 / 0.8 | 1.5 / 1 |  |
| Mouse 4 | 1 / 0.5 | 0.8 / 0.8 | 0.9 / 0.4 | 1 / 0.5 |  |
| Mouse 5 | 0.5 / 0.5 | 0.7 / 0.5 | 0.6 / 0.3 | 1 / 1 |  |

FMT, fecal microbiota transplantation; FMT-0d, Day 0 after FMT; FMT-4d, Day 4 after FMT; P group, mice receiving psoriatic microbiota transplantation; N group, mice receiving healthy microbiota transplantation; C group, mice receiving PBS; Mean (SD), Mean and SD of PASI scores (scales plus erythema).

**Supplementary Table S6. The number of epidermal cell layers in the back skin of mice psoriasiform models measured by three** **independent researchers** **under two high-power fields of light microscope.**

|  | Number of epidermal cell layers under one/another high-power fields (researcher 1) | Number of epidermal cell layers under one/another high-power fields (researcher 2) | Number of epidermal cell layers under one/another high-power fields (researcher 3) | Mean (SD) |
| --- | --- | --- | --- | --- |
| **Pre-FMT (P group)** |  |  |  | 9.967 (1.608) |
| Mouse 1 | 10 / 9 | 11 / 10 | 8 / 8 |  |
| Mouse 2 | 10 / 8 | 8 / 11 | 12 / 11 |  |
| Mouse 3 | 12 / 9 | 8 / 9 | 14 / 11 |  |
| Mouse 4 | 9 / 11 | 12 / 11 | 9 / 8 |  |
| Mouse 5 | 9 / 11 | 12 / 11 | 9 / 8 |  |
| **Pre-FMT (N group)** |  |  |  | 9.933 (1.552) |
| Mouse 1 | 10 / 9 | 11 / 10 | 8 / 9 |  |
| Mouse 2 | 10 / 8 | 8 / 11 | 12 / 11 |  |
| Mouse 3 | 12 / 9 | 8 / 9 | 14 / 12 |  |
| Mouse 4 | 9 / 11 | 12 / 11 | 9 / 8 |  |
| Mouse 5 | 9 / 11 | 8 / 9 | 10 / 10 |  |
| **Pre-FMT (C group)** |  |  |  | 9.933 (1.285) |
| Mouse 1 | 10 / 9 | 11 / 10 | 8 / 9 |  |
| Mouse 2 | 10 / 8 | 8 / 11 | 12 / 12 |  |
| Mouse 3 | 9 / 11 | 12 / 11 | 9 / 9 |  |
| Mouse 4 | 9 / 11 | 12 / 11 | 9 / 10 |  |
| Mouse 5 | 9 / 11 | 8 / 9 | 10 / 10 |  |
| **FMT-0d (P group)** |  |  |  | 7.567 (0.9714) |
| Mouse 1 | 7 / 7 | 7 / 8 | 8 / 9 |  |
| Mouse 2 | 7 / 8 | 8 / 9 | 8 / 8 |  |
| Mouse 3 | 6 / 8 | 6 / 7 | 9 / 8 |  |
| Mouse 4 | 8 / 9 | 9 / 8 | 7 / 6 |  |
| Mouse 5 | 6 / 8 | 6 / 7 | 8 / 7 |  |
| **FMT-0d (N group)** |  |  |  | 4.700 (0.8367) |
| Mouse 1 | 4 / 4 | 5 / 5 | 4 / 4 |  |
| Mouse 2 | 3 / 4 | 4 / 5 | 4 / 4 |  |
| Mouse 3 | 4 / 4 | 4 / 5 | 4 / 4 |  |
| Mouse 4 | 5 / 5 | 6 / 5 | 5 /6 |  |
| Mouse 5 | 6 / 5 | 6 / 6 | 5 /6 |  |
| **FMT-0d (C group)** |  |  |  | 5.167 (1.020) |
| Mouse 1 | 4 / 4 | 4 / 6 | 6 / 5 |  |
| Mouse 2 | 7 / 5 | 6 / 7 | 6 / 6 |  |
| Mouse 3 | 6 / 5 | 4 / 4 | 6 / 5 |  |
| Mouse 4 | 5 / 4 | 5 / 4 | 3 / 4 |  |
| Mouse 5 | 6 / 6 | 6 / 5 | 6 / 5 |  |
| **FMT-4d (P group)** |  |  |  | 4.167 (0.9855) |
| Mouse 1 | 2 / 5 | 4 / 5 | 3 / 4 |  |
| Mouse 2 | 4 / 4 | 4 / 5 | 6 / 5 |  |
| Mouse 3 | 4 / 4 | 4 / 3 | 3 / 3 |  |
| Mouse 4 | 3 / 4 | 3 / 4 | 4 / 4 |  |
| Mouse 5 | 5 / 6 | 4 / 6 | 5 / 5 |  |
| **FMT-4d (N group)** |  |  |  | 2.533 (0.7303) |
| Mouse 1 | 2 / 2 | 2 / 1 | 2 / 2 |  |
| Mouse 2 | 2 / 3 | 2 / 2 | 3 / 2 |  |
| Mouse 3 | 2 / 2 | 3 / 4 | 2 / 3 |  |
| Mouse 4 | 4 / 4 | 3 / 3 | 2 / 3 |  |
| Mouse 5 | 3 / 2 | 3 / 3 | 2 / 3 |  |
| **FMT-4d (C group)** |  |  |  | 2.433 (0.5683) |
| Mouse 1 | 2 / 2 | 2 / 2 | 2 / 2 |  |
| Mouse 2 | 2 / 3 | 3 / 3 | 2 / 3 |  |
| Mouse 3 | 4 / 3 | 3 / 2 | 3 / 3 |  |
| Mouse 4 | 2 / 2 | 3 / 2 | 3 / 2 |  |
| Mouse 5 | 2 / 2 | 2 / 3 | 2 / 2 |  |

FMT, fecal microbiota transplantation; FMT-0d, Day 0 after FMT; FMT-4d, Day 4 after FMT; P group, mice receiving psoriatic microbiota transplantation; N group, mice receiving healthy microbiota transplantation; C group, mice receiving PBS; Mean (SD), Mean and SD of number of epidermal cell layers under one high-power fields.

**Supplementary Table S7. The number of IL-17A^+^ cells in lesion of mouse psoriasiform models.**

|  | PFM-Pre | NFM-Pre | CON-Pre | PFM- 0d | NFM-0d | CON-0d | PFM- 4d | NFM- 4d | CON- 4d |
| --- | --- | --- | --- | --- | --- | --- | --- | --- | --- |
| M 1 | 21 | 33 | 18 | 21 | 15 | 19 | 16 | 8 | 14 |
| M 2 | 17 | 19 | 34 | 24 | 16 | 19 | 14 | 17 | 16 |
| M 3 | 34 | 30 | 33 | 26 | 19 | 18 | 12 | 13 | 11 |
| M 4 | 33 | 24 | 19 | 24 | 16 | 15 | 19 | 14 | 14 |
| M 5 | 26 | 26 | 27 | 24 | 17 | 18 | 16 | 14 | 13 |
| Mean SD | 26.2 7.396 | 26.4 5.413 | 26.2 7.530 | 23.8 1.789 | 16.6 1.517 | 17.8 1.643 | 15.4 2.608 | 13.2 3.271 | 13.6 1.817 |

PFM, mice receiving psoriatic fecal microbiota transplantation; NFM, mice receiving healthy fecal microbiota transplantation; CON, mice receiving PBS; PRE, Pre-fecal-microbiota-transplantation; 0d, at Day 0 after fecal microbiota transplantation; 4d, at Day 4 after fecal microbiota transplantation; M n, n mice in the same group.

**Supplementary Table S8. The number of IL-17A^+^ cells in gastrointestinal tissues of mouse psoriasiform models.**

|  | M 1 | M 2 | M 3 | M 4 | M 5 | Mean (SD) |
| --- | --- | --- | --- | --- | --- | --- |
| PFM-Pre | 7 | 4 | 5 | 5 | 6 | 5.4  (1.14) |
| NFM-Pre | 5 | 5 | 5 | 6 | 6 | 5.4 (0.5477) |
| CON-Pre | 5 | 5 | 4 | 6 | 6 | 5.2 (0.8367) |
| PFM- 0d | 12 | 13 | 18 | 19 | 16 | 15.6  (3.05) |
| NFM-0d | 7 | 8 | 4 | 5 | 6 | 6  (1.581) |
| CON-0d | 6 | 7 | 6 | 7 | 7 | 6.6  (0.5477) |
| PFM- 4d | 11 | 12 | 13 | 11 | 12 | 11.8  (0.8367) |
| NFM- 4d | 4 | 6 | 7 | 5 | 5 | 5.4  (1.14) |
| CON- 4d | 5 | 4 | 5 | 6 | 5 | 5  (0.7071) |

PFM, mice receiving psoriatic fecal microbiota transplantation; NFM, mice receiving healthy fecal microbiota transplantation; CON, mice receiving PBS; PRE, Pre-fecal-microbiota-transplantation; 0d, at Day 0 after fecal microbiota transplantation; 4d, at Day 4 after fecal microbiota transplantation; M n, n mice in the same group.

**Supplementary Table S9. Gut microbial alterations in patients with psoriasis/psoriatic arthritis compared with normal controls, based on published literatures.**

| Disease | Gut microbiota alterations | Methods | Ref. |
| --- | --- | --- | --- |
| Psoriasis | Reduced bacterial diversity; Decrease in abundance of *Coprococcus* species, *Parabacteroides*, unclassified *Ruminococcaceae*, *Akkermansia*, genera *Coprobacillus*, *Bacteroidetes*, and *Lachnospiraceae*. | 16S rRNA gene pyrosequencing technology | 13 |
| Psoriasis | A significantly higher variability; Reduced genus *Bacteroides*; Increased *Akkermansia* spp and Faecalibacterium. | 16S rRNA gene pyrosequencing technology | 33 |
| Psoriasis | Perturbed ratio of *Firmicutes* and *Bacteroidetes*; Underrepresented *Actinobacteria*. | RT-PCR | 34 |
| Psoriasis | Decreased phylum *Verrucomicrobia*, phylum *Tenericutes,* classs *Mollicutes*, class *Verrucomicrobiae,* order *Verrucomicrobiaes*, order *RF39*, family *Verrucomicrobiaceae*, family S24-7, genus *Akkermansia*, and *Akkermansia muciniphila*; Increased family *Bacteroidaceae*, family *Enterococcaceae*, genus *Enterococcus*, genus *Bacteroides*, and *Clostridium citroniae*. | 16S rRNA gene pyrosequencing technology | 16 |
| Psoriasis | Lower community richness; Reduced phylum *Firmicutes*, genus *Thermus*, *Streptococcus*, *Rothia*, *Granuli-catella*, *Gordonibacter*, *Allobaculum*, and *Carnobacterium*; Increased phylum *Bacteroidetes*, genus *Bacillus*, *Bacteroides*, *Bacteroidia*, *Sutterella*, *Lactococcus*, *Lachnospiraceae_UCG004*, *Lachnospira*, *Mitochondria_norank*, *Cyanobacteria_norank*, and *Parabacteroides*. | 16S rRNA gene pyrosequencing technology | 18 |
| Psoriasis | Decrease of *Faecalibacterium prausnitzii* together with an increase of *Escherichia coli*. | Quantitative PCR | 35 |
| Psoriasis | Lower microbial diversity; Increased phylum *Actinobacteria*, phylum *Firmicutes*, family *Bifidobacteriaceae, Coriobacteriaceae, Lachnospiraceae, Clostridiales*_Family XIII, *Eggerthellaceae, Peptostreptococcaceae, Ruminococcaceae, Erysipelotrichaceae*, genera *Blautia, Bifidobacterium, Collinsella, Slackia, Ruminococcus and Subdoligranulum*; Reduced phylum *Bacteroidetes*, phylum *Proteobacteria*, family *Bacteroidaceae, Barnesiellaceae, Prevotellaceae, Tannerellaceae, Burkholderiaceae, Rikenellaceae, Lactobacillaceae, Streptococcaceae, Desulfovibrionaceae, Veillonellaceae, Marinifilaceae, Victivallaceae, Pasteurellaceae*, genera *Bacteroides, Parabacteroides, Barnesiella, Alistipes, Paraprevotella*, and *Faecalibacterium*; *Akkermansia* did not show variability among groups. | 16S rRNA gene pyrosequencing technology | 14 |
| Psoriasis | Similar microbial diversity; Increased *Firmicutes* : *Bacteroides* ratio, *Actinobacteria* proportion, phylum *Firmicutes*, genera *Blautia*, *Faecalibacterium*, *Ruminoccocus* *gnavus* proportion, *Dorea formicigenerans* proportion, *Collinsella aerofaciens* proportion; Reduced *Proteobacteria* proportion, phylum *Bacteroidetes*, genera *Prevotella*, *Prevotella copri*. | 16S rRNA gene pyrosequencing technology | 15 |
| Psoriasis | Similar microbial diversity; Increased phylum *Synergistetes*, class *Synergistia*, order *Synergistales*, family *Veillonellaceae*, *Ruminococcaceae*, *Dethiosulfovibrionaceae*, genera *Faecalibacterium*, *Megamonas*, *Pyramidobacter*, *Gemmiger*; Reduced family *Lachnospiraceae*, *Fusobacteriaceae*, *Bacteroidaceae*, genera *Fusobacterium*, *Bacteroides*. | 16S rRNA gene pyrosequencing technology | 36 |
| Psoriasis | Similar microbial diversity and Firmicutes/Bacteroides ratio; Increased family *Ruminococcaceae*, genera *Faecalibacterium*; Reduced family *Lachnospiraceae*, genera *Oscillibacter*, *Roseburia*. | 16S rRNA gene pyrosequencing technology | 37 |
| Psoriasis | Similar species richness; Perturbed Firmicutes/Bacteroides ratio; Reduced phylum *Bacteroidetes*, class *Bacteroidia*, order *Bacteroidales*, family *Bacteroidaceae*, genera *Bacteroides*, *Paraprevotella*; Increased phylum *Firmicutes*, class *Clostridia*, order *Clostridiales*, genera *Faecalibacterium*, *Blautia*. | 16S rRNA gene pyrosequencing technology | 38 |
| Psoriasis | Similar microbial diversity; Increased phylum *Firmicutes*, family *Ruminococcaceae*, *Lachnospiraceae*, genera *Ruminococcus*, *Megasphaera*, *Dialister*, *Bifidobacterium*, *Dorea*, *Desulfovibrio*, *Alkanindiges*, *Mitsuokella* and *Collinsella*; Reduced phylum *Bacteroidetes*, family *Bacteroidaceae*, *Prevotellacea*, genera *Sutterella* and *Paraprevotella*, *Haemophilus*, *Veillonella* and *Clostridium*. | 16S rRNA gene pyrosequencing technology | 17 |
| Psoriasis | Significantly different Firmicutes/Bacteroidetes ratio; Reduced Actinobacterial phyla. | Fecal RT-PCR testing | 39 |
| Psoriatic Arthritis | Reduced bacterial diversity, Decrease in abundance of *Coprococcus* species, *Akkermansia*, *Ruminococcus*, *Pseudobutyrivibrio*, unclassified *Clostridia*, *Verrucomicrobia*, *Verrucomicrobiae*, *Verrucomicrobiales*, *Parabacteroides*, unclassified *Ruminococcaceae*, and *Alistipes*. | 16S rRNA gene pyrosequencing technology | 13 |
